# Diamines as switchable-hydrophilicity solvents with improved phase behaviour†

**DOI:** 10.1039/c8ra05751f

**Published:** 2018-07-31

**Authors:** Jesse R. Vanderveen, Jialing Geng, Susanna Zhang, Philip G. Jessop

**Affiliations:** Department of Chemistry, Queen's University 90 Bader Lane Kingston Ontario Canada K7L 3N6 jessop@queensu.ca

## Abstract

Removing solvents by distillation is not a sustainable process because it requires the use of volatile solvents and a high energy input. An alternative is to use a switchable-hydrophilicity solvent (SHS), which can be removed from products and recycled without any distillation step. SHSs are solvents that reversibly switch between hydrophilic and hydrophobic forms with the addition and removal of a trigger such as CO_2_. Monoamine SHSs can be separated from dissolved products by extraction into carbonated water, but the solvent removal is limited by the distribution coefficient of the SHS between the carbonated water phase and the product phase. In this article, the use of diamines as SHSs with improved distribution coefficients is explored. Several diamine SHSs are identified and their properties compared to those of monoamine SHSs. Comparisons include the p*K*_aH_ (the p*K*_a_ of the conjugate acid of a base) and log *K*_ow_ (log of the octanol–water partition coefficient) requirements for amines to act as SHSs, distribution coefficients, removal from hydrophobic liquids, switching speeds, and risks to the environment and human health and safety.

## Abstract 2

Diamine switchable-hydrophilicity solvents can be removed from products, by carbonated water, with much greater efficiency than past switchable solvents.

## Graphical abstract

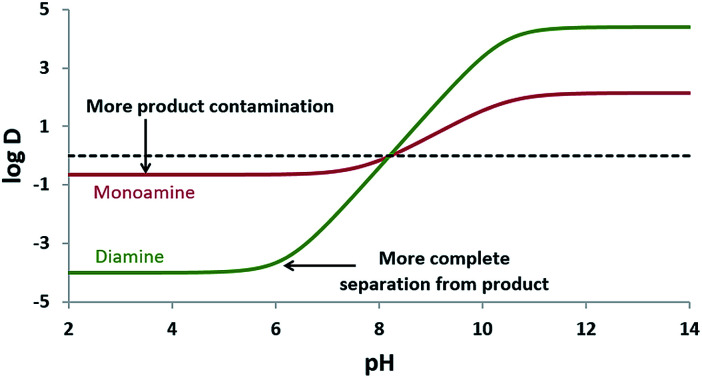

## Introduction

The traditional way of removing organic solvents from products in industry is distillation, but distillation is not a sustainable process because of two major drawbacks. First, distillation requires the use of volatile solvents, which are typically flammable and contribute to photochemical smog formation^1^ and inhalation risks to workers. Second, distillation has a large energy demand. Despite these drawbacks, volatile solvents are still preferred because distillation is a simple and effective method for separating solvents from their solutes.

One alternative to distillation for solvent–solute separations is a switchable-hydrophilicity solvent (SHS) based separation.^1–4^ SHSs are solvents that can reversibly switch between two different forms: a hydrophilic form that is miscible with water and a hydrophobic form that creates a biphasic mixture with water. The SHSs reported to date are hydrophobic amines or amidines that are poorly miscible with water in their neutral forms but can be protonated to make a water-soluble salt. The addition of CO_2_ into a biphasic SHS/water mixture converts the SHS from a hydrophobic neutral molecule into a hydrophilic bicarbonate salt and thereby creates a monophasic liquid mixture (eqn. (1)). The mixture can be made biphasic again by removing CO_2_ from the system.

1 NR_3(aq)_ + H_2_O_(l)_ + CO_2(g)_ ⇌ HNR_3(aq)_^+^ + HCO_3(aq)_^−^

**Fig. 1:**
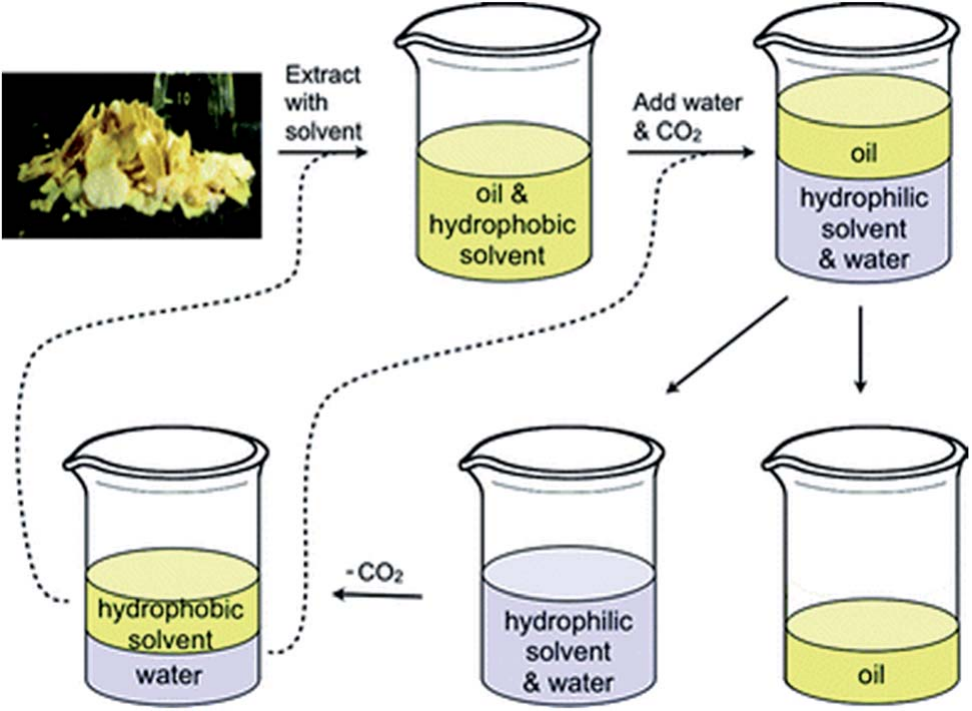
The process in which an SHS is used to extract oil from soybean flakes. The SHS and water are regenerated at the end of the cycle and can be reused (*via* the dashed lines) to process more soybean flakes. Reproduced from Jessop *et al.*, 2010 with permission from the authors.^2^

One of the most studied uses of SHSs is for the extraction and subsequent isolation of hydrophobic products, such as the extraction of lipids from soybeans (Fig. 1).^2^ In this process, the hydrophobic SHS is used to extract lipids from the soybeans, then the SHS is converted to its hydrophilic form by the action of water and CO_2_, causing it to dissolve in the aqueous phase. The lipids remain as a separate phase and can be isolated by decantation. Afterwards, the SHS is recovered from the aqueous phase by removing the CO_2_. The SHS and water are separated by decantation and both are reused in subsequent cycles of the process. In addition to extracting lipids from soybeans, SHSs have been used to isolate lipids from algae,^5–8^ bitumen from oil sands,^9,10^ and phenols from lignin pyrolysis oil.^11^ Interest has also been shown in applying SHSs to other applications such as water purification,^12–18^ microextraction for detecting and quantifying environmental contaminants,^19–25^ separations of reaction products from organocatalysts,^26^ emulsion formation,^27^ and processing recyclable materials.^3,28^

Previous studies showed that monoamine SHSs can be largely separated from dissolved products by the action of carbonated water, but some solvents might not be sufficiently removed from the product because the partitioning of the SHS into the aqueous phase was inadequate. One recent example of this incomplete separation is in the removal of SHS from low density polyethylene reported by Samorì *et al.*, where the recovered polyethylene was contaminated with up to 4 wt% SHS.^28^ A study of the liquid–liquid phase equilibria of one SHS-based system demonstrates that the extent of this contamination varies depending on the mass ratios of water, SHS, and product.^29^ Incomplete removal of SHS from the product leads to a loss of solvent and contamination of the product.

We hypothesized that diamine SHSs might be more readily separated from hydrophobic products than monoamine SHSs. In comparison to monoamines, which can be protonated to form a monocationic species, diamines can be protonated twice to form a more hydrophilic dicationic species. These diamine bis(bicarbonate) salts may partition more completely into the aqueous phase than monoamine bicarbonate salts, resulting in a more pure hydrophobic product phase. A study on switchable aqueous ionogens, a different type of CO_2_-switchable material that can increase or decrease the ionic strength of aqueous solutions, showed that diamine additives are retained in the aqueous phase better than monoamine additives in the presence of CO_2_.^30^ This enhanced partitioning into water may be a property of diamine SHSs as well and would be a beneficial property in the context of SHS-based separations such as the process shown in Fig. 1 by decreasing SHS contamination in the product.

In the present work, diamine SHSs are reported for the first time with 5 examples. Their partitioning between aqueous and organic phases as well as their ability to be separated from toluene (as a representative hydrophobic liquid product) are compared to those of monoamine SHSs. The properties required for a diamine to display switchable hydrophilicity are discussed. Finally, diamine SHSs are compared with monoamine SHSs and two common nonswitchable solvents to gain insight into the risks and benefits of diamine SHSs in terms of environmental, health, and safety hazards.

## Results and discussion

### Identification of diamine SHS

**Scheme 1:**
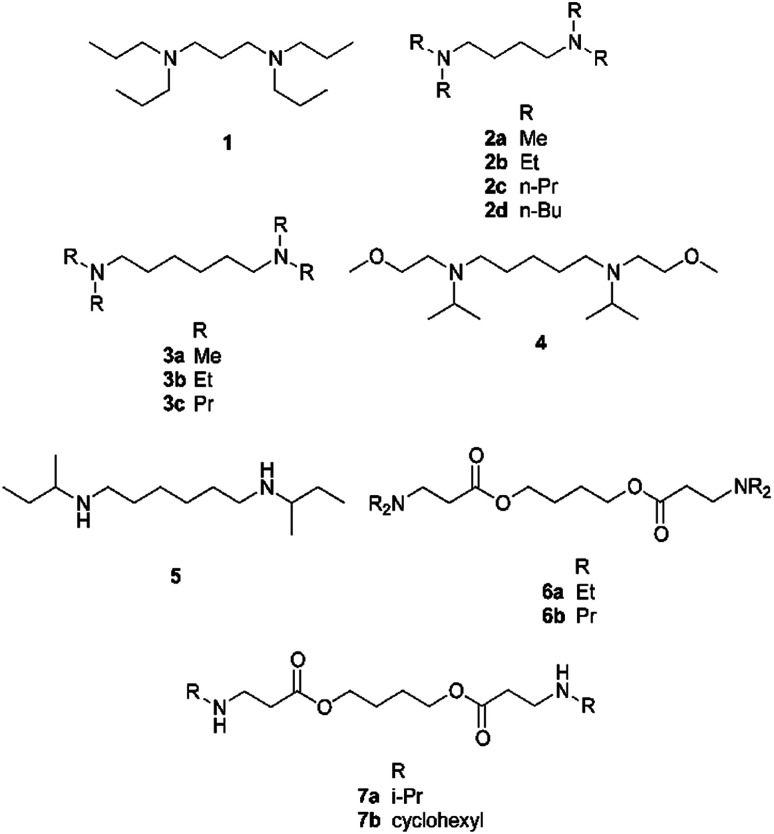
Diamines used in this study.

Fourteen diamines were tested for SHS behaviour (Table 1 and Scheme 1). A compound is considered to have SHS behaviour if it passes three tests. First, it must create a biphasic mixture with water (typically an equal volume of water) in the absence of CO_2_. The CO_2_ content in normal air is too low to affect the result. Second, the mixture from the first test must become monophasic upon exposure to 1 bar of CO_2_. Third, the mixture from the second test must revert to a biphasic mixture when CO_2_ has been thoroughly removed from the system.

**Table 1:** The behaviours of diamines when mixed with an equal volume of water or carbonated water as well as their predicted log *K*_ow_, p*K*_aH1_, and p*K*_aH2_ values

Behaviour	Compound	log *K*_ow_^a^	p*K*_aH1_^b^	p*K*_aH2_^b^
Monophasic	2a	0.7	10.0	8.5
Monophasic	3a	1.7	10.1	9.3
Monophasic	7a	2.2	9.5^c^	8.5^c^
Switchable	2b	2.7	10.8	10.0
Switchable	2c	4.7	10.2	9.3
Switchable	3b	3.7	10.9	10.2
Switchable	4	2.5	9.3	8.6
Switchable	7b	4.9	9.5^d^	8.5^d^
Biphasic	1	4.2	10.2	7.5
Biphasic	2d	6.6	10.2	9.3
Biphasic	3c	5.6	10.2	9.6
Biphasic	6a	2.8	9.0^c^	8.1^c^
Biphasic	6b	4.7	9.0^d^	8.1^d^
Precipitates	5	4.1	11.2	10.6

a Predicted using EPISUITE (KOWWIN v1.68) software.

b Predicted using Advanced Chemistry Development's ACD/Percepta v12.0 software, unless otherwise specified.

c Experimentally determined (this work).

d Estimated from experimental values of an analogous compound.

The compounds tested in this study were selected to have specific log *K*_ow_ (log of the octanol–water partition coefficient) and p*K*_aH1_ (p*K*_a_ of the monoprotonated species) values. The log *K*_ow_ and p*K*_aH_ values of monoamines were shown in past work to be determining factors for switchable hydrophilicity.^31–33^ The length of the hydrocarbon link between the two amine groups was also varied to determine how short it can be without preventing the compound's switchable behaviour. The proximity of the amine groups has an effect on the compound's p*K*_aH2_ (p*K*_a_ of the diprotonated species).^34^ When two equivalent amine groups are closely connected, p*K*_aH2_ is much lower than p*K*_aH1_, but when the two groups are distant, p*K*_aH2_ is only about 0.8 units below p*K*_aH1_. If a compound's p*K*_aH2_ is too small, it will not be sufficiently di-protonated by CO_2_ in water and will therefore not exhibit a sufficient change in hydrophilicity. Aqueous p*K*_aH_ values are used here because the acid–base switching reaction takes place in the presence of an aqueous phase and even though many of the amines are poorly miscible with water, most of the conjugate acids of the amines are likely to be soluble in water. Therefore, aqueous p*K*_aH_ is the most appropriate measure of basicity in this context.

The phase behaviours of these 14 compounds when mixed with water are shown in Table 1. The behaviours are reported for 1 : 1 (v/v) mixtures of water : diamine under air or CO_2_. Three compounds were too hydrophilic and were completely miscible with water, even in the absence of CO_2_ (“monophasic”). Five compounds formed biphasic mixtures with water, even in the presence of CO_2_ (“biphasic”). These compounds are either too hydrophobic to enter the aqueous phase as salts (*e.g.* compound 2d) or not basic enough and therefore not sufficiently protonated by CO_2_ to switch (*e.g.* compounds 6a and 6b). Compound 5 is poorly miscible with water in the absence of CO_2_, but it does not form a monophasic liquid when CO_2_ is added. Instead, the mixture solidifies (“precipitates”). The amine precipitates as a salt and water is likely either incorporated in the solid as a hydrate or trapped inside the solid because no liquids were visible in the sample. This behaviour has been observed before for monoamine SHSs and the precipitate in those cases was identified as an ammonium bicarbonate salt.^31,35^ Finally, five compounds displayed the desired switchable behaviour: poorly miscible with water in the absence of CO_2_ and completely miscible with water in the presence of CO_2_ (“switchable”).

The predicted octanol–water partition coefficient (log *K*_ow_) of every diamine in Table 1 and the experimental or predicted acidity of their conjugate acids (p*K*_aH1_ and p*K*_aH2_) were considered in order to determine the log *K*_ow_ and p*K*_aH_ requirements for diamines to act as SHSs. These values are listed in Table 1. A graph of this data relating log *K*_ow_ and p*K*_aH1_ to the switchable behaviour is available in the ESI.† The mean absolute error of the log *K*_ow_ predictions is stated to be 0.36 and this value is supported by our analysis (see ESI†). It appears that successful diamine SHSs have log *K*_ow_ values between approximately 2 and 5. Diamines such as compounds 2a, 3a, and 7a with lower log *K*_ow_ values are too hydrophilic to be SHSs because they form monophasic mixtures with water under air. Diamines such as compounds 3c and 2d with a log *K*_ow_ > 5 are too hydrophobic to be SHSs because they form biphasic mixtures with water under CO_2_.

If an amine does not have sufficient basicity, it will not be sufficiently protonated in the presence of CO_2_ to become hydrophilic. Based on the experimental and predicted p*K*_aH1_ values for diamines used in this study, a p*K*_aH1_ value of 9.5 is sufficient, but a value of 9.0 is not. Thus, compounds 6a and 6b are not SHSs, but compound 7b is. Predicted p*K*_aH1_ values were used for most diamines because the predictions indicated that the p*K*_aH1_ values are significantly greater than the cutoff value of p*K*_aH1_ = 9.5. For diamines with p*K*_aH1_ values near 9.0–9.5 (compounds 6a, 6b, 7a, and 7b), experimental p*K*_aH1_ and p*K*_aH2_ values were determined. The experimental p*K*_aH1_ and p*K*_aH2_ values for compound 4 could not be determined experimentally (see the experimental methods section in the ESI†), so its predicted p*K*_aH1_ and p*K*_aH2_ values were used. Compound 4 has a predicted p*K*_aH1_ value of 9.3 and it is an SHS. Therefore, the minimum p*K*_aH1_ value required for a diamine to be an SHS may be slightly lower than 9.5, or the predicted p*K*_aH1_ for compound 4 may differ from its real value by 0.2 units (the uncertainty of the prediction was ±0.4, see ESI†). Because the uncertainty of predicted p*K*_aH1_ values is relatively large, the predicted p*K*_aH1_ value of compound 4 was not used to determine the acceptable p*K*_aH1_ range. The upper limit of the p*K*_aH1_ range was not determined because amines typically do not have p*K*_aH_ values much higher than 11 in water.

These acceptable log *K*_ow_ and p*K*_aH1_ ranges apply to systems that contain a 1 : 1 (v/v) mix of water : diamine and are switched between ∼0 atm and ∼1 atm of CO_2_. Diamines that are too hydrophobic (log *K*_ow_ > 5) or not basic enough (p*K*_aH1_ < ∼9.5) to act as SHSs under these conditions may act as SHSs if more water or a higher pressure of CO_2_ is used.^33^

In addition to log *K*_ow_ and p*K*_aH1_, the effect of the alkyl chain length between the two nitrogen atoms was considered. The degree of separation between the two protonatable sites of a diamine affects the basicity of the second protonatable site.^34^ If the sites are too close, the p*K*_aH2_ value will be too low, the second site may not be sufficiently protonated by CO_2_, and the compound may not become water-miscible under a CO_2_ atmosphere. Compounds 1, 2c, and 3b all fit the log *K*_ow_ and p*K*_aH1_ requirements discussed above and should all display switchable hydrophilicity. However, compound 1 does not act as an SHS. It has a shorter chain length (3 carbons) connecting the nitrogen atoms than compounds 2c and 3b (4 and 6 carbons, respectively) and a lower p*K*_aH2_ (7.5 compared to 9.3 and 10.2, respectively) as a result. It appears that a diamine must not have a p*K*_aH2_ as low as 7.5 and therefore must have a minimum of 4 carbon atoms separating the two nitrogen atoms in order to act as an SHS. Again, the use of more water or a higher pressure of CO_2_ may allow for diamines with less separation of protonatable sites to act as SHSs.

The requirements for diamines to act as SHSs are different from those for monoamines. Our group has found that a monoamine must have a log *K*_ow_ between 1.2 and 2.5 to act as an SHS (using 1 bar of pressure and equal volumes of amine and water).^31,32^ It appears that diamines must be more hydrophobic than monoamines in order to act as SHSs. Wilson and Stewart proposed that tertiary amines containing only hydrocarbon functionalities (in addition to the amine group) should have a carbon : nitrogen ratio between 6 : 1 and 12 : 1 in order for it to display switchable miscibility with water.^12^ Based on the results of the hydrocarbon-functionalized diamines used in this study, the appropriate carbon : nitrogen ratio for diamines is between 6 : 1 (compound 2b) and 8 : 1 (compound 2c).

### Comparison of the switching process for monoamine and diamine SHS

**Fig. 2:**
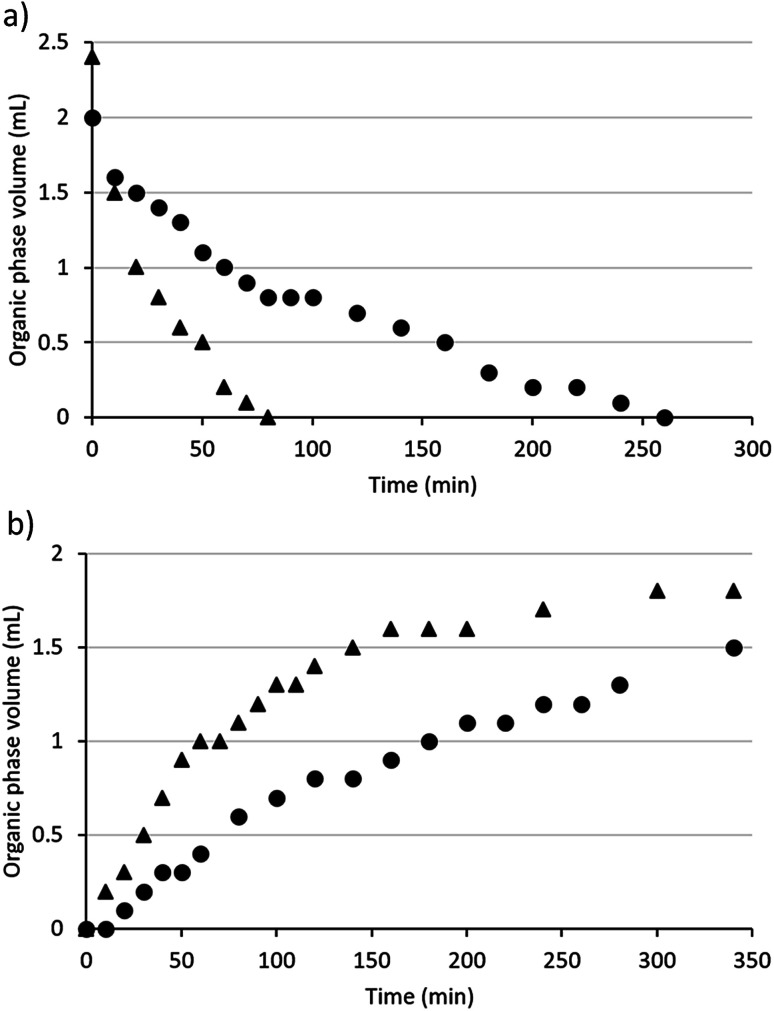
The volume of the organic phase in systems containing 2 mL water and 2 mL SHS (triangles = DMCA, circles = compound 3b) over time while bubbling gases through the mixture (a = CO_2_, b = Ar).

Diamine SHSs generally required more time than monoamines to switch between their hydrophobic and hydrophilic states (Fig. 2). The times required to switch diamine 3b and a monoamine SHS, *N*,*N*-dimethylcyclohexylamine (DMCA), were compared. When CO_2_ was passed through mixtures of each compound with water with the same volumes of SHS and water (2 mL each) and the same CO_2_ flow rate (10 mL min^−1^), it took DMCA 80 min to become monophasic with water but 260 min for diamine 3b to become monophasic with water. Although only 2 mL of DMCA was used, the initial volume of the organic phase was 2.4 mL due to the solubility of water in DMCA (21.4 wt% at 20.0 °C).^36^ Note that equipment designed to accelerate transfer of gases into aqueous solutions was not used but would likely accelerate the switching process.

DMCA also separated from water faster than diamine 3b at 60 °C with argon bubbled through the mixture at 15 mL min^−1^. Although both SHSs began to separate from water within 20 min under these conditions, the organic phase increased in volume faster in the case of DMCA than it did for compound 3b. After heating and argon-bubbling continued overnight, the volume of the organic phase containing compound 3b was restored to 2 mL the next day while the aqueous phase had decreased to 1.4 mL, indicating the loss of water from the system. When the experiment continued overnight for the system containing DMCA, the volume of the organic phase was only 1.7 mL as opposed to the 2.4 mL that would be expected and the volume of the aqueous phase was 1.6 mL. Thus the diamine SHS switches successfully in both directions but somewhat more slowly than the monoamine SHS.

Another difference between monoamine and diamine SHSs is in their tendency to form a foam while gases are being bubbled through their mixtures with water. Mixtures of diamine SHSs and water created substantially more foam than mixtures of monoamine SHSs and water. In the context of switching speeds, the conditions described above were used to limit foaming for diamine 3b to simplify quantification of volumes and to prevent the loss of material due to foam overflowing the vessel. Differences in gas flow rates can affect the switching speeds of SHSs. For example, DMCA has been reported to become monophasic with an equal volume of water within 16 min using a CO_2_ flow rate of 100 mL min^−1^.^3^ Therefore, both diamines and monoamines can be switched faster than is represented in Fig. 2 if the conditions are changed, but monoamines are expected to switch faster than diamines under the same conditions.

### Comparison of monoamine and diamine SHS distribution between aqueous and organic phases

**Fig. 3:**
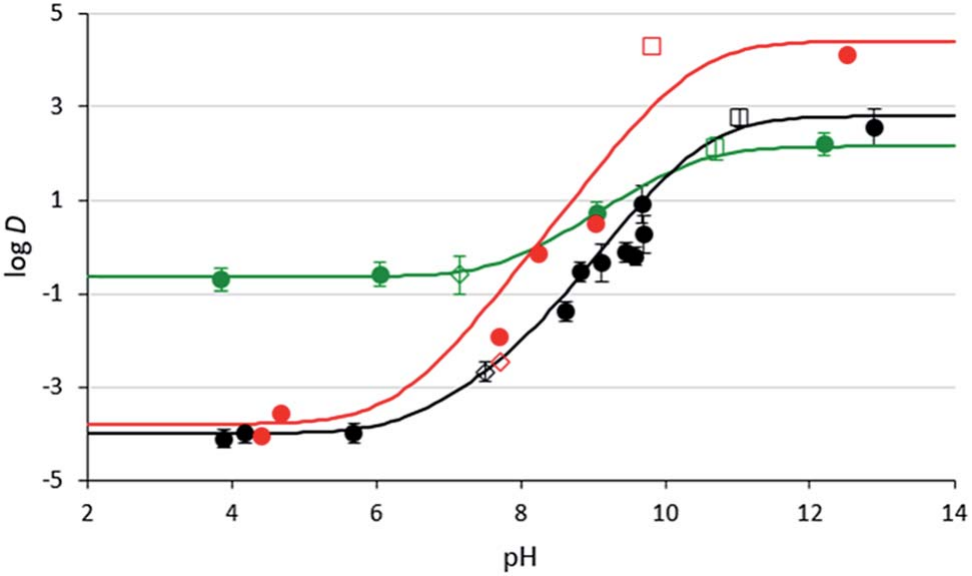
1-Octanol/water distribution coefficients of compounds 3b (black) and 2c (red) at room temperature at varying aqueous pH values. pH values were either not adjusted (empty squares), adjusted with CO_2_ (empty diamonds), or adjusted with glycolic acid or NaOH (filled in circles). Values for compound 3b were measured in triplicate and the error bars are shown. The lines represent the log *D* curve estimated from the data (see ESI for details†). The data and estimated curve for DMCA (green) were taken from a previous study.^29^

In order to determine whether diamine SHSs can be more completely removed from hydrophobic phases than monoamine SHSs, log *D* values (log of the distribution coefficient) were measured to determine how an SHS would distribute between water (aqueous phase) and 1-octanol (representing a typical organic phase) at different pH values (Fig. 3). log *D* is similar to log *K*_ow_, but it includes the conjugate acids and bases of a compound in its measurement. Therefore, log *D* changes as a function of pH because the amounts of protonated and unprotonated species changes with pH. On the other hand, log *K*_ow_ is not a function of pH because it only considers the neutral species. log *D* and log *K*_ow_ are equivalent for non-ionizable compounds and also for ionizable compounds at pH values where the concentration of the ionic species is negligible. A low distribution coefficient (log *D* ≪ 0) suggests that the species tends to partition more into the aqueous phase; a high distribution coefficient (log *D* ≫ 0) suggests that the species tends to partition more into the organic phase. An ideal SHS would have a high distribution coefficient in its neutral form at high pH conditions and a low distribution coefficient in its charged form at low pH conditions. Overall, a SHS with good switchable behaviour would have a large change in log *D* (Δ log *D*) between protonated and unprotonated forms. This behaviour would improve the separation of SHS from a dissolved product when CO_2_ and water are added to the mixture (upper right section of Fig. 1).

The distribution coefficients, *D*, of compounds 2c and 3b at different pH levels have been measured and compared to literature distribution coefficients for DMCA.^32^ In their fully protonated forms, compounds 2c and 3b exhibit log *D* values of −3.8 and −4.0 respectively, compared to −0.7 for DMCA. These results suggest that diamine SHSs partition more favourably into the aqueous phase in their fully protonated forms than monoamine SHSs, causing them to separate more completely from a hydrophobic material when CO_2_ is introduced. This property would result in more pure isolated hydrophobic product as well as more complete reusability of the SHS since less SHS would be lost by association with the isolated hydrophobic product. In their neutral forms, compounds 2c and 3b exhibit log *D* values of 4.1 and 2.6 respectively, compared to 2.1 for DMCA, which means that in basic conditions, diamines partition more favourably into the organic phase than monoamines. Overall, compounds 2c and 3b have much larger Δ log *D* between carbonated and uncarbonated solutions (Δ log *D* = 7.9 and 6.6, respectively) than DMCA (Δ log *D* = 2.8). The fact that Δ log *D* is dramatically larger for diamine SHSs could create significant process advantages.

The liquid–liquid equilibria of systems containing diamine 3b, toluene, and carbonated water under CO_2_ were also studied and compared to similar reported systems using DMCA.^29^ In these systems, toluene acted as a representative hydrophobic liquid product that was to be isolated. To determine the extent to which the toluene is contaminated with SHS, the mass ratio of SHS/toluene in the organic phase was determined (Table 2). The data confirm that contamination of the hydrophobic product (toluene) is lower with the diamine than the monoamine, but only when the total amount of SHS is small. In mixtures with lower fractions of SHS, the toluene is contaminated with approx. 0.1 wt% diamine 3b compared to approx. 4 wt% DMCA, representing an approx. 40-fold decrease in contamination. In mixtures with higher fractions of SHS, however, diamine 3b and DMCA contaminate the toluene to similar extents because the higher pH in the presence of the diamine prevents the log *D* of the diamine from being sufficiently low.

**Table 2:** Comparison of the contamination of toluene with monoamine (DMCA) and diamine (compound 3b) SHSs in systems with different compositions of SHS, water, and toluene at 30.0 °C and under 1 atm CO_2_

System composition SHS : water : toluene (mass percent)	SHS in organic phase (g SHS/g toluene)		Aqueous pH	
	Compound 3b	DMCA^a^	Compound 3b	DMCA^a^
42 : 31 : 27	0.66 ± 0.006	0.69 ± 0.004	9.05 ± 0.05	8.75 ± 0.05
27 : 39 : 34	0.22 ± 0.003	0.16 ± 0.003	8.99 ± 0.05	8.51 ± 0.05
13 : 47 : 40	0.0015 ± 0.001	0.037 ± 0.002	8.05 ± 0.05	8.11 ± 0.05

a Data from ref. 28.

The effects of pH on the effective separation of diamine SHSs from hydrophobic liquids is also demonstrated by these results. Diamine 3b showed no advantage over DMCA in mixtures with aqueous pH values near 9.0, but it did show an advantage over DMCA at an aqueous pH value near 8.0. This result is in agreement with the log *D* curve shown in Fig. 3, where the log *D* values of diamine 3b and DMCA differ much more at pH ≤ 8 than at pH ≥ 9. Therefore the combination of diamine SHS with an elevated CO_2_ pressure might be attractive for applications using larger % loading of SHS.

### Comparison of monoamine and diamine SHSs for the extraction of lipids from soybeans

One of the proposed applications of SHSs is the extraction of lipids from soybeans as outlined in Fig. 1.^2^ This extraction and separation process was performed once using diamine 3b and again using DMCA. The amount of lipids extracted in both cases was 14 wt%. A similar extraction using hexanes also recovered 14 wt% lipids from the soybeans. The ^1^H NMR spectra of these extracts are similar but the spectrum of the extract obtained from DMCA has additional signals, indicating the presence of impurities that may make the use of DMCA less desirable (see ESI, Fig. S2†).

The expected benefit of the diamine SHSs over the monoamine SHSs is in the purity of the recovered lipids. An analysis of the lipids using gas chromatography revealed that DMCA was present in the soybean oil at concentrations of 20 g L^−1^, while diamine 3b was only present at 1 g L^−1^. These results demonstrate that a diamine SHS can be more completely removed from a hydrophobic product than a monoamine SHS without using additional water.

### Hazard comparison of monoamine and diamine SHSs

SHSs are of interest because they can be separated from solutes without distillation, so solvent volatility and flammability can be minimized without impacting the energy requirements for separation. However, a lack of volatility is not sufficient to make a solvent safe. A solvent should be as safe as possible for humans and the environment in addition to performing its task well. Therefore, diamine SHSs should be compared and contrasted with monoamine SHSs and other solvents in terms of their impacts on human health and safety as well as their impact on the environment.

A comparison of monoamine and diamine SHSs in terms of several properties related to health, safety, and environmental hazards is presented in Table 3. While experimental measurements of toxicity are outside of the scope of the project, predicted data give an indication of whether the toxicity or other effects are expected to be severe, moderate, or mild. Values were predicted using the U.S. Environmental Protection Agency's Toxicity Estimation Software Tool v4.1 (TEST). Experimentally determined values for each compound are included where available. Four diamine SHSs are included in the table along with two trialkylamine SHSs, a monoamine SHS designed to have few hazards (di-(4-methoxybutyl)isopropylamine, “DMBIPA”), and toluene and hexanes as commonly used solvents. Diamine 7b was not included in this comparison because it is hydrolytically unstable due to its ester functionalities and therefore not suitable for use as a solvent in SHS-based applications.

**Table 3:** Properties related to environmental, health, and safety hazards for diamine SHSs, monoamine (DMCA, triethylamine, and DMBIPA) SHSs, toluene and hexanes, calculated from TEST. Experimental values are included in brackets where available^a^

Compound	Fathead minnow LC_50_ (mg L^−1^)	*Daphnia magna* LC_50_ (mg L^−1^)	Oral rat LD_50_ (mg kg^−1^)	Boiling point (°C) at 1 atm	Vapour pressure (mTorr) at 25 °C	Flash point (°C)	Bioaccumulation factor
2b	64	4.0	400	226 (230)^b^	15	79	17
2c	3.3	2.5	630	270 (240)^b^	3	117	86
3b	42	4.9	340	249 (280)^b^	40	96	29
4	150	4.0	3000	318 (310)^b^	6.8 × 10^−2^	145	55
DMCA	100	38	320 (348)	159 (160)	3100 (52 000)^d^	38 (42)	9.6
Triethylamine	470	140	1300 (460)	85 (89)	62 000 (57 000)	5 (−15)^d^	4.1 (1.6)
DMBIPA	97	11	1600	254 (270)^c^	1	103	32
Toluene	35 (34)	30 (98)	1100 (636)	125 (110)	23 000 (28 000)	22 (10)	49
*n*-Hexane	11 (2.5)	49	3500 (25 000)	73 (69)	130 000 (151 000)	−3 (−26)^d^	171

a Experimental values were obtained from the TEST database unless stated otherwise.

b Extrapolated from boiling point under reduced pressure determined in this study using a nomograph.

c Extrapolated from a reported boiling point under reduced pressure reported using a nomograph.^37^

d Data from a safety data sheet provided by Sigma-Aldrich.

Diamines 2b, 2c, and 3b are less volatile than monoalkylamine SHSs and therefore have higher (safer) flash points. However, they are also predicted to be more toxic toward fish and invertebrates because they have lower predicted fathead minnow and *Daphnia magna* LC_50_ values. Finally, these diamine SHSs are comparable to monoamine SHSs in terms of predicted LD_50_ values and bioaccumulation factors. In comparison to toluene and *n*-hexane, these diamine SHSs are much less volatile, are nonflammable, but are likely to have higher acute oral toxicity and higher toxicity towards *Daphnia magna*.

Unlike the other diamine SHSs, diamine 4 was designed to minimize environmental, health, and safety hazards and so it is discussed separately here. This compound was identified using a virtual screening process similar to a process developed for monoamine SHSs.^37^ For details on this process, see the ESI.† Diamine 4 is preferable to every other diamine SHS in every category except *Daphnia magna* LC_50_ and bioaccumulation factor, where it is comparable to the other diamine SHSs. Rather than comparing diamine 4 to trialkylamine SHSs, it is more appropriate to compare it to DMBIPA, which is a monoamine SHS that was designed to minimize hazards using the virtual screening process. Diamine 4 and DMBIPA are comparable in every property in Table 3. The difference in *Daphnia magna* LC_50_ values are not likely to be significant given the uncertainty associated with the predicted values. Diamine 4 is safer with regards to volatility (boiling point, flash point, and vapour pressure), but DMBIPA itself has a low volatility compared to most common solvents and so the benefit of further decreased volatility offered by diamine 4 may not be as important as other concerns. Considering the common solvents, diamine 4 is predicted to be as safe as or safer than toluene and *n*-hexane in all hazards considered in Table 3 except for *Daphnia magna* LC_50_, where it is expected to be more hazardous.

One environmental concern that is not prevalent for monoamine SHSs but may be for diamine SHSs is bioaccumulation. According to the GHS, a bioconcentration factor (BCF) less than 500 indicates low levels of bioaccumulation but log *K*_ow_ can be used as a surrogate measure of bioaccumulation with log *K*_ow_ < 4 indicating low levels of bioaccumulation. Bioaccumulation is not a concern for monoamine SHSs because they typically do not have log *K*_ow_ values greater than 2.5.^3,31–33^ However, the log *K*_ow_ range required for diamines to act as SHSs is approximately 2–5 and so some diamine SHSs might have the potential to bioaccumulate. Despite the possibility of having high log *K*_ow_ values, diamine SHSs are not likely to bioaccumulate. It has been shown that the BCF of ionizable compounds depend on the pH of the environment, with bases having lower BCFs at lower pH,^38^ and that log *D* may be a better surrogate for bioaccumulation than log *K*_ow_.^39^ Given the p*K*_aH1_ and p*K*_aH2_ values of the diamine SHSs, they are likely to have low log *D* values in environments with pH < 9 and therefore have low risk of bioaccumulation. This hypothesis is supported by the predictions for diamines made by TEST; the predicted bioaccumulation factors are all less than 100.

It is important to remember that most of the values listed in Table 3 are based on predictions and that the actual values may differ. An evaluation of the prediction accuracy of TEST for amines has been performed in previous studies of monoamine SHSs.^31,37^ Some predictions, such as boiling and flash points, are reasonably accurate (usually within 10 °C of the experimental value), but other predictions are not as accurate. For example, predicted LD_50_ (oral, rat) values for amines were found to be within a factor of 3 of the experimental values 95% of the time. Although these predictions have low precision, they can still be used to distinguish more dangerous compounds from less dangerous compounds if the values differ by an order of magnitude. Experimental quantification of these values was considered outside the scope of this study but would be a valuable exercise if these solvents are considered for large scale use.

## Conclusions

The first CO_2_-switchable diamine SHSs were identified and these SHSs were compared to monoamine SHSs. Diamines require larger log *K*_ow_ values to be SHSs than monoamines. The range of C : N atom ratios required for SHS with only alkyl functionalities is narrower for diamines than for monoamines. Diamine SHSs partition more favourably into water than monoamine SHSs if the pH of the aqueous phase is sufficiently low (pH ∼8). A liquid–liquid equilibrium study showed that a diamine SHS partitioned more completely into the aqueous phase than a monoamine SHS. This enhanced separation from hydrophobic products was also demonstrated in an extraction of lipids from soybeans, where the amount of residual SHS in the extracted lipids was much lower when a diamine SHS rather than a monoamine SHS was used.

Two results disfavour the use of diamines. First, the time required for diamine SHSs to switch between their hydrophobic and hydrophilic states is longer than for monoamine SHSs. Second, diamine SHSs tend to produce foam during the switching process, a phenomenon that does not occur significantly when switching monoamine SHSs.

The safety of diamines and monoamines was also considered using predicted values. In general, some, but not all, diamine SHSs are predicted to have greater ecotoxicity to *Daphnia magna* but, more favourable, higher flash points than monoamine SHSs. A virtual screening approach can be used to identify diamine SHSs that are predicted to have reduced risks, such as diamine 4.

## Conflicts of interest

The corresponding author, P. G. J., holds patents on switchable-hydrophilicity solvents.

## Supplementary Material

RA-008-C8RA05751F-s001
