# Supplementary material for: Diamines as switchable-hydrophilicity solvents with improved phase behaviour
Source: RSC Adv. 2018 Jul 31;8(48):27318–25. doi: 10.1039/c8ra05751f (PMC9083370; doi:10.1039/c8ra05751f)
Supplement: RA-008-C8RA05751F-s001 [file RA-008-C8RA05751F-s001.pdf]

Electronic Supplementary Material (ESI) for RSC Advances.
This journal is © The Royal Society of Chemistry 2018

Supplementary Information for

Diamines as switchable-hydrophilicity solvents with improved phase behaviour

Jesse R. Vanderveen, Jialing Geng, Susanna Zhang, and Philip G. Jessop

Department of Chemistry, Queen's University, 90 Bader Lane, Kingston, Ontario, K7L 3N6, Canada.
Email: jessop@queensu.ca

Contents:

1. Experimental methods
2. Graphical comparison of diamine switchability with log K_{ow} an pK_{aH1}
3. ^{1}H NMR spectra of soy flake extracts
4. Comparison of predicted and experimental log K_{ow} and pK_{aH} values
5. Virtual screening process for identifying diamine SHS
6. References
7. ^{1}H NMR spectra of synthesized compounds

1. Experimental Methods

All chemicals were used as received. The synthesis and characterization of the diamines used in this study are discussed in the supplementary information. Diamines **2a** and **3a** were purchased from TCI and Sigma-Aldrich, respectively. Glycolic acid, potassium carbonate, and *N*,*N*-dimethylcyclohexylamine were purchased from Sigma-Aldrich. CO_2 (4.8, supercritical fluid chromatography grade) and argon (4.8) were purchased from Praxair.

Synthesis and characterization information for diamines 6a, 6b, 7a, and 7b

The following procedure was adapted from the procedure for conversion of mono-acrylates by Zou and Jiang.[1] A mixture of 2 mL (10.6 mmol) 1,4-butanediol diacrylate and 40 mmol of a primary or secondary amine was stirred overnight at room temperature in a round bottom flask. Afterwards, the product was purified by heating the mixture to 50 °C under vacuum for 4 h to remove volatile compounds. No further purification was performed.

1,4-Butanediol di-(3-(diethylamino)propanoate) (6a) Using 4.1 mL diethylamine. Isolated yield: 95%. ^{1}H NMR (499.12 MHz, $CDCl_3$): δ = 1.01 (t, *J* = 7.1 Hz, 12H), 1.71 (broad triplet, 4H), 2.42 (t, *J* = 7.3 Hz, 4H), 2.53 (q, *J* = 7.1 Hz, 8H), 2.79 (t, *J* = 7.3 Hz, 4H), 4.11 (broad triplet, 4H); ^{13}C NMR (125.50 MHz, $CDCl_3$) 11.8, 25.3, 32.3, 46.8, 48.1, 63.8, 172.8; ν_{max} (ATR-FTIR) cm^{-1}: 744, 1172, 1466, 1732 (C=O), 2806, 2968; HRMS (ESI): calculated for $C_{18}H_{37}O_4N_2$ (MH^+): 345.27478, found: 345.27420.

1,4-Butanediol di-(3-(dipropylamino)propanoate) (6b) Using 5.5 mL dipropylamine. Isolated yield: 95%. ^{1}H NMR (499.12 MHz, $CDCl_3$) δ = 0.85 (t, 12H, *J* = 7.4), 1.42 (sextet, 8H, *J* = 7.4), 1.71 (broad triplet, 4H), 2.34 (t, 8H, *J* = 7.4), 2.41 (t, 4H, *J* = 7.3), 2.75 (t, 4H, *J* = 7.3), 4.10 (broad triplet, 4H); ^{13}C NMR (125.50 MHz, $CDCl_3$): δ = 11.8, 20.3, 25.3, 32.5, 49.5, 55.9, 63.7, 172.9; ν_{max} (ATR-FTIR) cm^{-1}: 745, 1048, 1188, 1239, 1461, 1735 (C=O), 2805, 2873, 2958; HRMS (EI): calculated for $C_{22}H_{45}O_4N_2$ (MH+): 401.33738, found: 401.33700.

1,4-Butanediol di-(3-(isopropylamino)propanoate) (7a) Using 3.5 mL isopropylamine. Isolated yield: 95%. ^{1}H NMR (499.12 MHz, $CDCl_3$): δ = 1.07 (d, *J* = 6.3 Hz, 12H), 1.39 (b, 2H), 1.71 (broad triplet, 4H), 2.49 (t, *J* = 6.6 Hz, 4H), 2.82 (septet, *J* = 6.3 Hz, 2H), 2.89 (t, *J* = 6.6 Hz, 4H), 4.12 (broad triplet, 4H); ^{13}C NMR (125.50 MHz, $CDCl_3$) δ = 22.9, 25.2, 34.9, 42.5, 48.4, 63.8, 172.7; ν_{max} (ATR-FTIR) cm^{-1}: 745, 1170, 1469, 1729 (C=O), 2871, 2961, 3326 (N-H); HRMS (ESI): calculated for $C_{16}H_{33}O_4N_2$ (MH^+): 317.24348, found: 317.24282.

1,4-Butanediol di-(3-(cyclohexylamino)propanoate) (7b) Using 4.6 mL cyclohexylamine. Isolated yield: 95%. ^{1}H NMR (499.12 MHz, $CDCl_3$): δ = 1.03 (qt (apparent), 4H), 1.15 (tt, J^1 = 12.3 Hz, J^2 = 6.1 Hz, 2H), 1.23 (qt, J^1 = 12.3 Hz, J^2 = 6.8 Hz, 4H) 1.31 (b, 2H), 1.61 (dt, J^1 = 12.3 Hz, J^2 = 6.8 Hz, 2H) 1.64-1.80 (m, 8H), 1.93 (d, *J* = 12.5 Hz, 4H), 2.42 (tt, J^1 = 10.4 Hz, J^2 = 3.7 Hz, 2H), 2.48 (t, *J* = 6.6 Hz), 2.92 (t, *J* = 6.6 Hz), 4.11 (broad triplet, 4H), ^{13}C NMR (125.50 MHz, $CDCl_3$): δ = 25.0, 25.3, 26.1, 33.6, 35.1, 42.1, 56.5, 63.8, 172.8; ν_{max} (ATR-FTIR) cm^{-1}: 744, 1167, 1449, 1730 (C=O), 2851, 2923, 3326 (N-H); HRMS (ESI): calculated for $C_{22}H_{41}O_4N_2$ (MH^+): 397.30608, found: 397.30527.

Synthesis and characterization information for diamines 2b, 2c, and 2d

Succinyl chloride was converted to *N*,*N*,*N′*,*N′*-tetraalkylsuccinamide following a procedure adapted from Peng *et al.* for conversion of mono-acyl chlorides.[2] A solution containing amine (100 mmol) in 55 mL dichloromethane was stirred at 0 °C. A second solution containing succinyl chloride (20 mmol) in 20 mL dichloromethane was added to the first solution dropwise over the course of 1 h. Afterwards, the mixture was allowed to warm to room temperature with stirring. The mixture was stirred overnight, then a 1 M aqueous solution of $HCl_{(aq)}$ (70 mL) was added and the product was extracted with 3 x 70 mL dichloromethane. The organic layer was dried with $MgSO_4$, filtered, and then concentrated under reduced pressure to yield the crude succinamide intermediate.

The reduction of the succinamide intermediate was performed following a procedure adapted from Knapick *et al.*[3] A mixture of lithium aluminium hydride (80 mmol) in 25 mL tetrahydrofuran was prepared under inert conditions in a 2-neck round bottom flask equipped with a reflux condenser. The crude succinamide intermediate was dissolved in 50 mL tetrahydrofuran and slowly added to the lithium aluminium hydride solution. The mixture was refluxed overnight, then cooled to 0 °C in an ice bath. Water was slowly added to the mixture until all of the remaining lithium aluminium hydride had reacted. The mixture was filtered via vacuum filtration and the filtrate was concentrated under reduced pressure. A 50 mL solution of 10% $NaOH_{(aq)}$ was added to the concentrated mixture, after which the product was extracted with 3 x 50 mL diethyl ether. The combined organic phase was dried with $MgSO_4$, filtered, and then concentrated under reduced pressure. The crude product was purified by vacuum distillation.

***N,N,N′,N′*-Tetraethyl-1,4-butanediamine (2b)** Using diethylamine. Isolated yield: 50%, b.p.: 63 °C (1 torr). ^{1}H NMR (499.12 MHz, $CDCl_3$): δ = 0.95 (t, 12H, *J* = 7.2 Hz), 1.39 (broad triplet, 4H), 2.38 (broad triplet, 4H), 2.47 (q, 8H, *J* = 7.2 Hz); ^{13}C NMR (125.50, $CDCl_3$): δ = 11.7, 20.31, 25.1, 46.8, 52.9; ν_{max} (ATR-FTIR) cm^{-1}: 765, 1069, 1202, 1292, 1381, 1468, 2795, 2871, 2933, 2967. *m/z* (EI): 200 (4) [M^+], 171 (13), 128 (7), 126 (10), 114 (4), 98 (30), 86 (100), 84 (15), 73 (15), 58 (26), 56 (16) ; HRMS (EI) calculated for $C_{12}H_{29}N_2$ (MH^+): 201.23253; found: 201.23297.

***N,N,N',N'*-Tetrapropyl-1,4-butanediamine (2c)** Using dipropylamine. Isolated yield: 55%, b.p. 50 °C (0.2 torr). ^{1}H NMR (499.12 MHz, $CDCl_3$): δ = 0.86 (t, *J* = 7.4 Hz, 12H), 1.36-1.54 (m, 12H), 2.36 (t, *J* = 7.5 Hz, 8H), 2.40 (broad triplet, 4H); ^{13}C NMR (125.50 MHz, $CDCl_3$): 12.0, 20.3, 25.1, 29.3, 54.2, 56.3; ν_{max} (ATR-FTIR) cm^{-1}: 746, 1076, 1190, 1378, 1463, 2796, 2871, 2933, 2956; *m/z* (EI): 256 (4) [M^+], 213 (4), 156 (10), 154 (27), 128 (29), 126 (23), 114 (100), 112 (37), 101 (10), 98 (11), 86 (8), 84 (21), 72 (32), 70, (15), 55 (7); HRMS (EI): calculated for $C_{16}H_{36}N_2$ (M^+): 256.2878; found: 256.2871.

***N,N,N',N'*-Tetrabutyl-1,4-butanediamine (2d)** Using dibutylamine. Isolated yield: 35%, b.p. 89 °C (0.2 torr) (lit. 107-108 °C at 0.3 mm Hg).[4] ^{1}H (499.12 MHz, $CDCl_3$) δ = 0.90 (t, *J* = 7.3 Hz, 12H), 1.30 (sextet, *J* = 7.3 Hz, 8H), 1.36-1.46 (m, 12H), 2.30-2.47 (m, 12H); ^{13}C NMR (125.50 MHz, $CDCl_3$) 14.1, 21.7, 25.1, 29.3, 53.9, 54.2; ν_{max} (ATR-FTIR) cm^{-1}: 732, 1084, 1179, 1376, 1465, 2795, 2861, 2929, 2954; *m/z* (EI): 312 (3), 255 (5), 184 (11), 182 (21), 142 (100), 128 (10), 126 (32), 112 (18), 100 (14), 98 (8), 92 (8), 86 (29), 84 (26), 71 (18), 57 (10), 55 (9); HRMS (EI): calculated for $C_{20}H_{44}N_2$ (M^+): 312.3504, found: 312.3508. This compound has been reported before but only information regarding its boiling point was presented.[4]

Synthesis and characterization information for diamines 1, 3b, 3c, and 5

A mixture containing 3 g of dibromoalkane and 8 equivalents of amine was stirred and refluxed overnight. The mixture was allowed to cool to room temperature, then 30 mL water was added to it and solid NaOH was dissolved in the water until the pH of the aqueous phase was >12. The product was extracted with 3 x 30 mL diethyl ether. The combined organic fractions were dried with $MgSO_4$, filtered, and then the diethyl ether was evaporated using a rotary evaporator to afford the crude product. The product was purified by vacuum distillation.

***N,N,N',N'*-Tetrapropyl-1,3-propanediamine (1)** Using 1,3-dibromopropane and dipropylamine. Isolated yield: 25%, b.p. 49 °C (0.2 torr) (lit. 120-130 °C at 10 torr).[5] ^{1}H NMR (499.12 MHz, $CDCl_3$): δ = 0.86 (t, *J* = 7.5 Hz, 12H), 1.43 (sextet, *J* = 7.5 Hz, 8H), 1.57 (p, *J* = 7.5 Hz, 2H), 2.35 (t, *J* = 7.5 Hz, 8H), 2.40 (t, J = 7.5 Hz, 4H); ^{13}C NMR (125.50 MHz, $CDCl_3$): δ = 12.0, 20.2, 24.7, 52.4, 56.3; ν_{max} (ATR-FTIR) cm^{-1}: 745, 1078, 1191, 1378, 1462, 2797, 2872, 2933, 2956; *m/z* (EI): 242 (3) [M^+], 141 (33), 126 (13), 114 (100), 112 (99), 98 (24), 86 (53), 72 (25), 70 (56), 58 (8), 56 (11); HRMS (EI): calculated for $C_{15}H_{35}N_2$ (MH^+): 243.27948, found: 243.27895. This compound has been reported before but only information regarding its boiling point was presented.[5]

***N,N,N',N'*-Tetraethyl-1,6-hexanediamine (3b)** Using 1,6-dibromohexane and diethylamine. Isolated yield: 80%. BP 80 °C (0.2 torr) (lit. 88 °C at 1 mm Hg).[6] ^{1}H NMR (499.12 MHz, $CDCl_3$): δ = 0.95 (t, 12H, *J* = 7.1 Hz), 1.24 (broad pentet, 4H, *J* = 6.9 Hz), 1.40 (broad pentet, 4H), 2.35 (t, 4H), 2.45 (q, 8H, *J* = 7.1 Hz); ^{13}C NMR (125.50 MHz, $CDCl_3$): δ = 11.70, 27.05, 27.73, 46.93, 53.02; ν_{max} (ATR-FTIR) cm^{-1}: 770, 1069, 1203, 1292, 1381, 1467, 2795, 2858, 2930, 2967; *m/z* (EI): 228 (43) [M^+], 199 (33), 142 (36), 112 (22), 86 (23), 58 (14); HRMS (EI): calculated for $C_{14}H_{33}N_2$ (MH^+): 229.26383, found: 229.26464. This compound has been reported before but only information regarding its boiling point was presented.[6]

***N,N,N',N'*-Tetrapropyl-1,6-hexanediamine (3c)** Using 1,6-dibromohexane and dipropylamine. Isolated yield: 40%, b.p. 90 °C (0.2 torr). ^{1}H NMR (499.12 MHz, $CDCl_3$) δ 0.87 (t, J=4 Hz, 12H), 1.28 (broad pentet, 4H), 1.35-1.52 (m, 12H), 2.30-2.45 (m, 12H); ^{13}C NMR (125.50 MHz, $CDCl_3$) δ 11.9, 20.2, 27.1, 27.6, 54.2, 56.3; ν_{max} (ATR-FTIR) cm^{-1}: 745, 1077, 1191, 1378, 1464, 2796, 2871, 2931, 2956; *m/z* (EI): 284 (44) [M$^+$], 256 (42), 241 (42), 182 (37), 154 (40), 114 (28), 86 (23); HRMS (EI): calculated for $C_{18}H_{41}N_2$ (MH$^+$): 285.32643, found: 285.32686.

***N,N'*-Di-*sec*-butyl-1,6-hexanediamine (5)** Using 1,6-dibromohexane and sec-butylamine. Isolated yield: 50%, b.p. 90 °C (1 torr) (lit. 95 °C at 0.5 torr)[7]. ^{1}H NMR (499.12 MHz, $CDCl_3$): δ = 0.89 (t, *J* = 7.5 Hz, 6H), 1.01 (d, *J* = 6.3 Hz, 6H), 1.20-1.40 (m, 6H), 1.40-1.55 (m, 6H), 2.53 (m, 4H), 2.60 (m, 2H); ^{13}C NMR (125.50 MHz, $CDCl_3$) 10.2, 19.9, 27.5, 29.6, 30.5, 47.4, 54.6; ν_{max} (ATR-FTIR) cm^{-1}: 710, 1099, 1164, 1372, 1461, 2806, 2854, 2925, 2960. *m/z* (EI): 228 (3) [M$^+$], 213 (4), 199 (41), 171 (7), 156 (7), 154 (24), 140 (11), 128 (24), 126 (100), 114 (25), 112 (4), 100 (25), 98 (61), 92 (7), 86 (51), 81 (7), 72 (12), 70 (12), 56 (21); HRMS (ESI): calculated for $C_{14}H_{33}N_2$ (MH$^+$): 229.26383, found: 229.26369. This compound has been reported before but only information regarding its boiling point was presented.[7]

Synthesis and characterization information for diamine 4

A mixture of 16 mL 2-bromo-1-methoxyethane (170 mmol) and 30 mL isopropylamine (370 mmol) was stirred and refluxed overnight. The mixture was cooled, 25 mL water was added, and solid NaOH was added until the pH of the water was >12. The mixture was extracted with 3 x 25 mL diethyl ether. The combined organic phase was dried with $MgSO_4$, filtered, and then concentrated under reduced pressure. The resulting liquid was distilled, isolating three fractions at temperatures of 52 °C, 93 °C, and 134 °C. The fractions were collected separately and analyzed by ^{1}H NMR spectroscopy. The third fraction contained the desired reaction intermediate (*N*-(2-methoxyethyl)isopropylamine, 9.6 g, 50% yield).

The reaction intermediate was used as the amine reactant in a reaction with glutaryl chloride, following the same procedure as that described above in Section 2. The resulting amide was reduced with lithium aluminium hydride, again following the procedure described in Section 2. The product was purified by distillation

***N,N'*-Dimethoxyethyl-*N,N'*-diisopropyl-1,5-pentanediamine (4)** Yield: 65%, BP 120 °C (1 torr). ^{1}H NMR (499.12 MHz, $CDCl_3$): 0.99 (d, *J* = 6.6 Hz), 12H), 1.26 (p, *J* = 7.6 Hz, 2H), 1.43 (p, *J* = 7.6 Hz, 4H), 2.40 (t, *J* = 7.6 Hz, 4H), 2.59 (t, *J* = 6.8 Hz, 4H), 2.93 (septet, *J* = 6.6 Hz, 2H), 3.35 (s, 6H), 3.40 (t, *J* = 6.8 Hz, 4H); ^{13}C NMR (125.50 MHz, $CDCl_3$): δ = 18.2, 25.3, 29.0, 49.3, 51.2, 51.4, 58.8, 72.7; ν_{max} (ATR-FTIR) cm^{-1}: 965, 1119, 1175, 1360, 1461, 2811, 2870, 2928, 2965; *m/z* (EI): 301 (1), 287 (3), 270 (5), 257 (100), 239 (239), 186 (37), 172 (8), 156 (11), 153 (23), 142 (95), 140 (53), 138 (17), 130 (53), 126 (15), 112 (38), 106 (17), 100 (54), 98 (51), 88 (31), 86 (11), 84 (16), 72 (10), 70 (12), 59 (24), 56 (24); HRMS: calculated for $C_{17}H_{39}N_2O_2$ (MH$^+$): 303.30060, found: 303.30105.

Testing diamines for switchable hydrophilicity

To test if a diamine displays switchable hydrophilicity, a mixture containing an equal volume of water and the diamine was prepared at room temperature, about 20 °C. If the resulting mixture was monophasic, the diamine was not considered an SHS. If the mixture was biphasic, CO_2 was bubbled into

the mixture using a gas dispersion tube (Ace Glass, 7 mm O.D., 25-50 µm porosity) until it became monophasic or for up to 6 h. If the mixture remained biphasic, the diamine was not considered an SHS. If the mixture became monophasic, CO_2 was removed by heating the mixture to 60 °C and then sparging it with argon using a gas dispersion tube (Ace Glass, 7 mm O.D., 25-50 µm porosity) until the mixture became biphasic again or for up to 6 h. If the mixture remained monophasic, the diamine was not considered to be an SHS. If the mixture became biphasic again, the diamine was considered to be an SHS.

Monitoring the switching of SHSs over time

A mixture of 2 mL water and 2 mL SHS (either DMCA or diamine **3b**) was prepared in a 10 mL graduated cylinder covered with a septum. The mixture was agitated briefly and then the volume of the organic phase was recorded. A needle was inserted into the septum as a vent and another needle was inserted into the septum and lowered into the SHS/water mixture. CO_2 was bubbled through the solution via the second needle at a rate of 10 mL/min, measured using an Intelligent Digital Flowmeter (Varian). The needle used to bubble CO_2 was removed from the mixture briefly at intervals to record the volume of the organic phase. The needle was inserted back into the mixture after the volume of the organic phase was recorded. The CO_2 bubbling was allowed to continue until no organic phase was visible.

After the organic phase disappeared, the CO_2 being bubbled through the solution was replaced with Ar (flow rate = 15 mL/min) and the graduated cylinder was placed in an oil bath heated to 60 °C. The needle used to bubble Ar was removed from the mixture and the graduated cylinder was raised out of the oil bath briefly at intervals to record the volume of the organic phase. The needle was inserted back into the mixture and the graduated cylinder was lowered back into the oil bath after the volume of the organic phase was recorded. The volume of the organic phase was monitored for 340 min, then Ar bubbling at 60 °C was continued overnight. The Ar bubbling and heating were stopped the next day and the final volume of the organic phase was recorded.

Measuring log *D* of diamine SHSs

For log *D* measurements, diamine (**2c** or **3b**, 0.50 mL) was added to a mixture of 5.0 mL water and 5.0 mL 1-octanol into a vial at room temperature. Glycolic acid, CO_2, or NaOH was added to the mixture to adjust the pH of the solution. For samples containing glycolic acid, the acid was added in specific molar equivalences relative to the diamine (diamine **3b**: 0.25, 0.50, 0.75, 1.0, 1.25, 1.5, 1.75, 2.0, 2.5, and 3.0 equivalents; diamine **2c**: 0.50, 1.0, 1.5, 2.0, and 3.0 equivalents). For samples containing CO_2, CO_2 was bubbled through the mixture at a rate of 50 mL min^{-1} for 1 h using a gas dispersion tube (Ace Glass, 7 mm O.D., 25-50 µm porosity). For samples containing NaOH, 1 molar equivalent of NaOH relative to the diamine was added to the mixture. The mixture was stirred overnight and allowed to settle for 2 h. The pH of the aqueous phase was measured using an Orion 4-Star benchtop pH-conductivity meter (Thermo Scientific) and then both the aqueous layer and the organic layer were sampled separately and diluted in a 5 mL volumetric flask with methanol as required for analysis (e.g. at high pH, the organic phase required substantial dilution, but the aqueous phase required very little). Butanol (20 µL) was added as an internal standard. The concentration of diamine in each solution was measured using GC-FID and used to calculate log *D* for the diamine at the measured pH. Samples for diamine **3b** were made in triplicate and averaged.

Measuring the amount of SHS in the toluene rich phase in the liquid-liquid equilibria of systems containing SHS, toluene, and carbonated water

Mixtures containing different mass fractions of diamine **3b**, water, and toluene were prepared to match the proportions of some of the mixtures previously studied that used DMCA instead of diamine **3b**.[8] CO_2 was bubbled through the mixtures for 8 h using a needle, then the mixtures were heated to 30.0 °C in a constant temperature bath for 16 h. After this time, the organic phase of each mixture was sampled and the amount of toluene and SHS in that phase was determined by GC-FID. The pH of the aqueous phase of each sample was measured using an Orion 4-Star benchtop pH-conductivity meter (Thermo Scientific). The experiments for each sample with different mass fractions were performed in triplicate and the averaged results were reported as the mass ratio of SHS:toluene in the organic phase of each mixture.

Extraction of lipids from soybeans using SHSs

To a 250 mL round bottom flask containing a magnetic stir bar was added 25 g of soy flakes and 50 mL of solvent (diamine **3b**, DMCA, or hexanes). The flask was sealed with a stopper and stirred vigorously for 18 h at room temperature (21 °C). Afterwards, the contents of the flask were transferred to a Büchner funnel and the liquid phase of the mixture was isolated by vacuum filtration through coarse porosity filter paper.

In the extraction using hexanes, the filtrate was transferred to a pre-weighed round bottom flask and the initial round bottom flask, the residual soy flakes, and the filtration flask were washed with a 50 mL portion of hexanes, which was also transferred to the pre-weighed round bottom flask. The hexanes were then evaporated under reduced pressure to afford the soybean extract.

In extractions using diamine **3b** or DMCA, the filtrate was transferred to a 500 mL separatory funnel containing 150 mL of water. CO_2 was bubbled through the mixture for 6 h using a gas dispersion tube (Ace Glass, 7 mm O.D., 25-50 µm porosity). The remaining organic phase was collected and then centrifuged to ensure more complete separation of the organic phase from the aqueous phase. After centrifugation, the organic phase (~3.5 g total mass) was collected using a syringe.

To ensure all of the SHS and lipids were transferred to the final mixture, all of the glassware and the soy flakes were washed with 50 mL hexanes, which was then evaporated under reduced pressure to afford a mixture of extract and SHS. The mixture was transferred to the separatory funnel. The mixture was washed with 25 mL water with CO_2 being bubbled through the mixture for 2 h. The resulting organic phase was then centrifuge and collected as described above.

A portion of each sample of soybean extract was dissolved in chloroform-*d* and analyzed by ^{1}H NMR spectroscopy. For samples extracted using SHS, residual SHS in the extract was measured by silica solid phase extraction of 1 mL extract, using 50 mL ethyl acetate to remove the lipids and then recovering the amine using 50 mL diethylamine. The diethylamine fraction was concentrated under reduced pressure, taken up into 1 mL methanol with 20 µL acetonitrile and analyzed by GC-FID.

Measuring pK_{aH1} and pK_{aH2}

The ionization constants of diamines **6a** and **7a** at room temperature (~20 °C) were determined using a procedure analogous to that described by Speakman for diprotic acids.[9] A known amount of diamine was dissolved in water and the solution was titrated with a 0.1 M solution of HCl standardized by titrating potassium carbonate. The pH of the solution was measured after every addition of HCl using an Orion 4-Star benchtop pH-conductivity meter (Thermo Scientific). The data was interpreted using the Fortran code described by Albert and Serjeant to determine the pK_a values of diprotic acids, adapted for analysis of dibasic compounds and translated to operate in MATLAB.[10] The pK_{aH} values for diamines **6b** and **7b** could not be accurately measured by this method due to their limited solubility in water. Their

values were assumed to be equivalent to those of diamines **6a** and **7a**, respectively. Diamine **4** was not sufficiently soluble in water to measure its pK_{aH} values accurately and no suitable similar molecule was identified to measure the values indirectly as was done for diamines **6b** and **7b**. The pK_{aH} values of the remaining diamines were not experimentally determined because their pK_{aH1} values were expected to be greater than those of the ester-containing diamines and, therefore, meet the pK_{aH1} requirement for displaying switchable hydrophilicity. Furthermore, many of these compounds are not likely to be sufficiently soluble in water to determine their pK_{aH} values using the method described above. The unmeasured pK_{aH} values were predicted using ACD/LABS Percepta software rather than being determined experimentally.

The accuracy of the predicted values from ACD/LABS Percepta software has been discussed previously.[11] The software itself reports a standard deviation of ±0.4 for each pK_{aH} value and the data collected previously supports this reported standard deviation.[11] Diamines with predicted pK_{aH1} values greater than 10 are expected to have experimental pK_{aH1} values greater than 9.5 and therefore have acceptable basicity to be SHS.

2. Graphical comparison of diamine switchability with log K_{ow} and pK_{aH1}

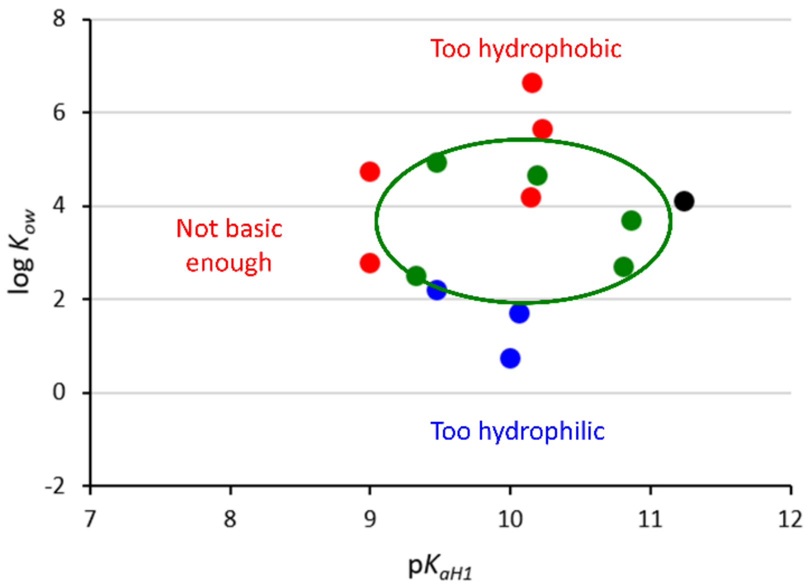

Fig. S1 A plot of log K_{ow} vs. pK_{aH1} of the diamines tested for switchable hydrophilicity from Table 1. Green dots represent diamines with switchable hydrophilicity. Red dots represent diamines that always form biphasic mixtures with water. Blue dots represent diamines that always form monophasic mixtures with water. Black dots represent diamines that form a solid when CO_2 is added. The area within the green oval shows the approximate range of log K_{ow} and pK_{aH1} values required for a diamine to be an SHS, although there is insufficient data to determine the upper limit of the range of acceptable pK_{aH1} values.

3. ^{1}H NMR spectra of soy flake extracts

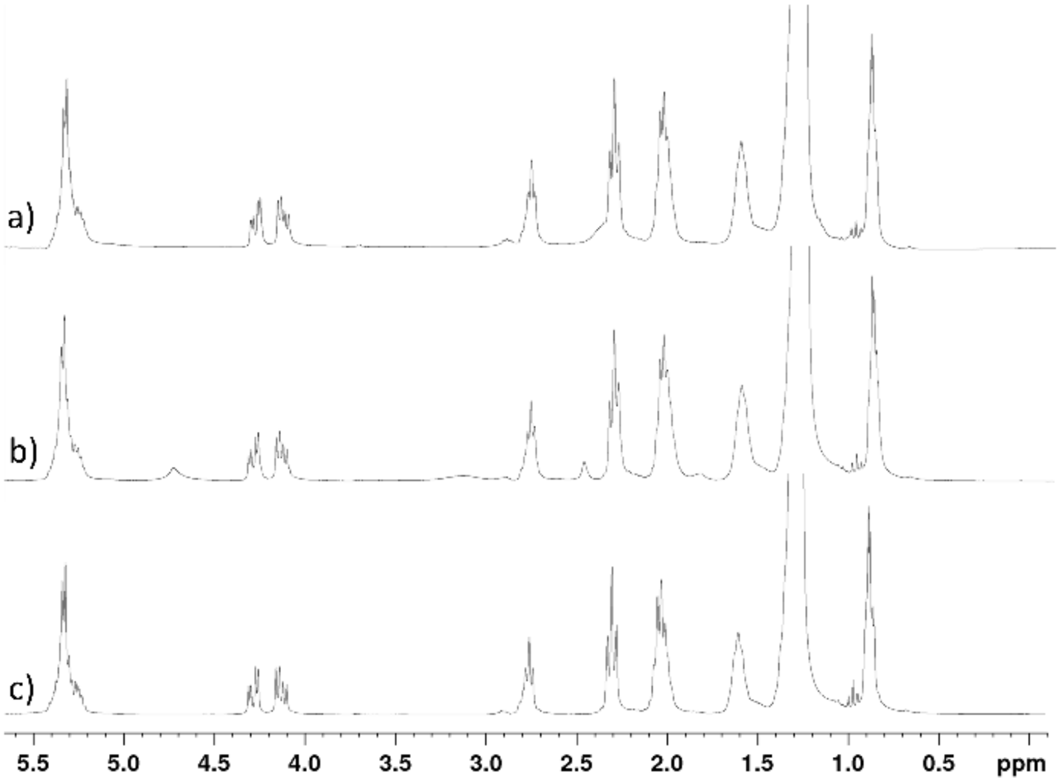

Fig. S2 ^{1}H NMR spectra in chloroform-*d* of soybean extract from extraction processes using a) diamine **3b**, b) DMCA, and c) hexanes.

4. Comparison of predicted and experimental log K_{ow} and pK_{aH} values

Predicted log K_{ow} and pK_{aH} values have been used throughout this study as stand-ins for experimentally determined values. Predicted values have been used successfully in the past to predict SHS behaviour.[11,12] A comparative analysis of the predicted and experimental pK_{aH} values of amines has been conducted previously and found that predicted pK_{aH} values from ACD/Percepta have a mean absolute error (MAE) of ~0.2 for amines (the reported MAE of ACD/Percepta is 0.4).[11] The difference between predicted and experimental pK_{aH1} values for compounds **6a** and **7a** were 0.4 and 0.2, respectively. The difference between predicted and experimental pK_{aH2} values for compounds **6a** and **7a** were 0.7 and 0.6, respectively. A plot of predicted vs. experimental pK_{aH} values of amines has been reproduced in Fig. S3.

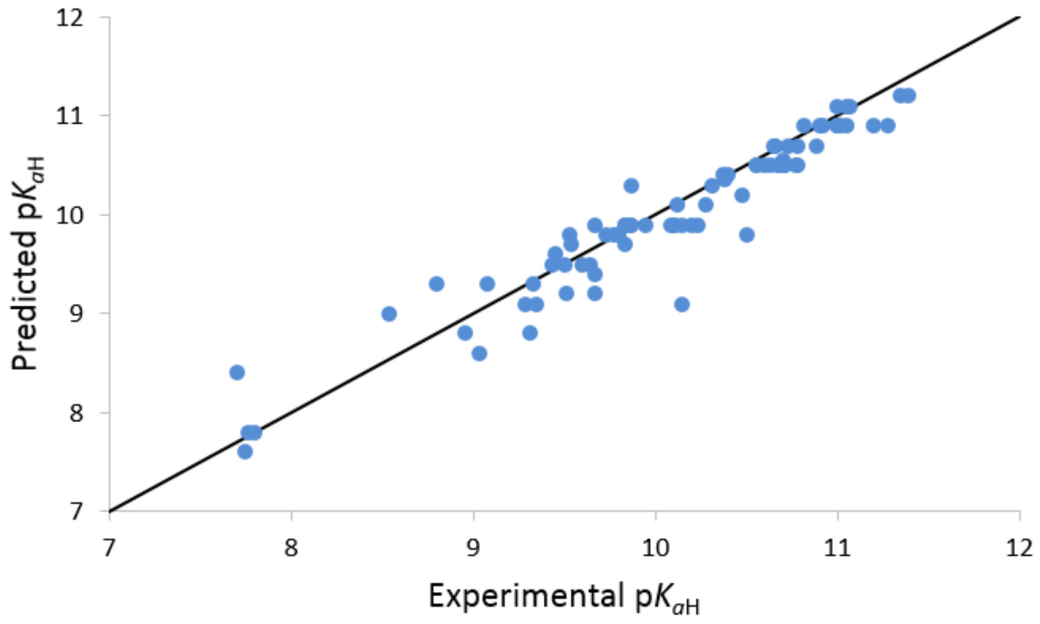

Fig. S3 A plot of predicted pK_{aH} values from ACD/Percepta vs. experimental pK_{aH} values of amines. This figure is reproduced from Vanderveen *et al.*, 2015, with permission from the authors.[11] The line represents a perfect prediction (predicted = experimental).

A comparative analysis of the predict and experimental log K_{ow} values has also been performed in the past, but not for the EPISUITE (KOWWIN v1.68) software.[11] Such a study has been performed for this research. A plot of predicted vs. experimental log K_{ow} values is shown in Fig. S4. The MAE for amines was calculated using equation S1, where n is the number of amines used in the comparison and f_i and y_i are the predicted and experimental values, respectively, for compound i. The dataset consists of thirty amines that may have one or more of the following functional groups: alcohol, ether, phenyl, and ketone. were used in the comparison and the MAE was found to be 0.3 (the reported MAE of KOWWIN v1.68 is 0.36).

$$MAE = \frac{1}{n}\sum_{i=1}^{n}|f_i - y_i| \qquad \text{Eqn. S1}$$

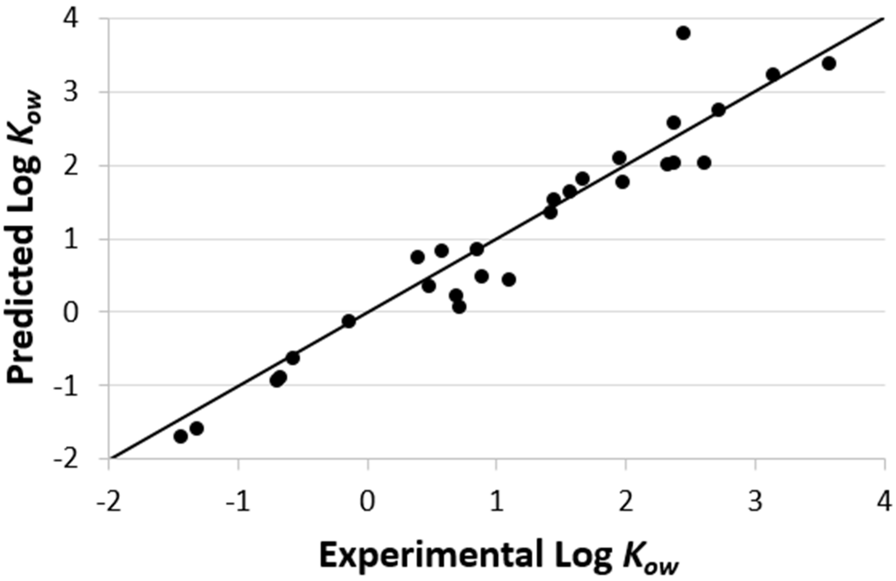

Fig. S4 A plot of predicted log K_{ow} values from KOWWIN v1.68 vs. experimental log K_{ow} values of amines. The line represents a perfect prediction (predicted = experimental).

5. Virtual screening process for identifying diamine SHS

Diamines that are likely to act as SHS and pose little risk to the environment or human health and safety were identified using the virtual screening approach developed by Vanderveen *et al.*[11]

A virtual combinatorial library of diamines was created using SmiLib v2.0.[13,14] Five central components were used, each containing two tertiary amine groups connected by an alkyl chain or alkyl ether chain (Fig. S5a). The central components were functionalized at positions 1-4 with substituents developed from the fragments shown in Fig. S5b. By functionalizing the carbon atoms with two fragments instead of three, quaternary carbon centres adjacent to the nitrogen, which can be synthetically challenging to make, can be avoided. Limiting the functionalization of the carbon atoms also prevents the software from generating excessively large numbers of structures. The substituents attach to the carbon atoms at position "A" and some can be extended at positions R^1 and R^2 with additional fragments. The substituents were limited to include a maximum of 8 heavy atoms (C, O or N) to prevent an overwhelming number of substituents from being created. Additionally, the two nitrogen atoms were substituted with the same 4 substituents so that they would be equivalent. This requirement was included to minimize the synthetic difficulty of the resulting compounds and to prevent the software from generating excessively large numbers of structures.

The log K_{ow} values for all of the molecules were predicted using EPISUITE (KOWWIN v1.68) software. The K_{ow} acceptability (A_{Kow}) of each compound, representing the likelihood that the predicted log K_{ow} corresponds to an experimental log K_{ow} that meets the design requirements for a diamine SHS, was calculated using either equation S2 or S3. Equation S2 assesses if a compound meets the lower log K_{ow} limit (2.0), while equation S3 assesses if a compound meets the upper log K_{ow} limit (5.0). These equations were developed to account for the deviation of the predicted values from the experimental values. The software user guide reports a MAE of ±0.36 and our limited dataset of amines showed a MAE of ±0.3.

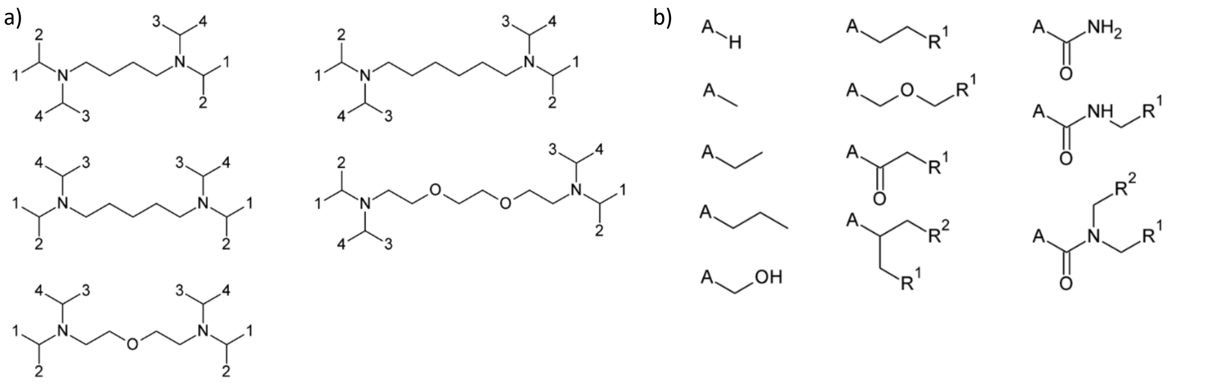

Fig. S5 a) Scaffold structures used to create the virtual combinatorial library. b) Fragment molecules used to create substituents to attach to the scaffold structures.

To account for this deviation, the predicted log K_{ow} of a series of amine-containing compounds were compared with their experimentally determined log K_{ow} values (Fig. S6). These differences were used to identify the 10th-90th percentile deviation between experimental and predicted values. Every 10th percentile deviation from the target value (log K_{ow} = 2.0 for the minimum log K_{ow} and log K_{ow} = 5.0 for the maximum log K_{ow}) was calculated. These "percentile log K_{ow}" values represent the log K_{ow} values that lie on the 10th – 90th percentile likelihood that a predicted value corresponds to an acceptable experimental value (one that meets or exceeds the target value). Each "percentile log K_{ow}" values was assigned an acceptability value between 0 and 1, incrementing by 1/8 every tenth percentile such that the 10th percentile gives A_{Kow} = 0, the 20th gives A_{Kow} = 0.125, the 30th gives A_{Kow} = 0.25, and so on until the 90th percentile gives A_{Kow} = 1. Assigning these acceptabilities to the "percentile log K_{ow}" values indicates that if a predicted value has less than a 10% chance of meeting the requirements, it is considered completely unacceptable; if a predicted value has greater than a 90% chance of meeting the requirement, it is considered completely acceptable. Intermediate predicted log K_{ow} values are considered acceptable to certain degrees. Equations S2 and S3 were derived by plotting each acceptability as a function of predicted log K_{ow} and fitting these points to a sigmoidal function. These data points and fit curves are shown in Fig. S7. The A_{Kow} of each compound was defined as the lowest value between equations S2 and S3.

$$A_{Kow} = \frac{1}{1+\left(\frac{25.17}{\log K_{ow}+23.02}\right)^{169.98}} \quad \text{Eqn. S2}$$

$$A_{Kow} = \frac{1}{1+\left(\frac{11.19}{\log K_{ow}+6.53}\right)^{-75.11}} \quad \text{Eqn. S3}$$

The compounds with A_{Kow} > 0.5 were then evaluated for pK_{aH1}. The pK_{aH1} values were predicted using Advanced Chemistry Development's ACD/Percepta v12.0 software. From these pK_{aH1} values, the pK_{aH} acceptability of each compound, A_{pKaH}, was calculated using equation S4, taken from Vanderveen *et al*.[11] This equation gives a quantitative measure of the probability that the predicted pK_{aH1} for a compound will be greater than or equal to 9.5.

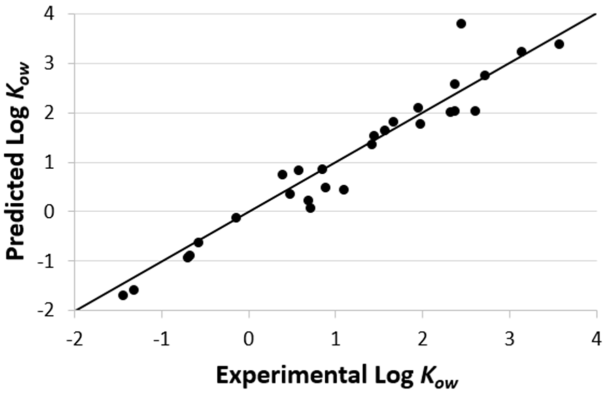

Fig. S6 A comparison of predicted and experimental log K_{ow} values, reproduced from Fig. S4.

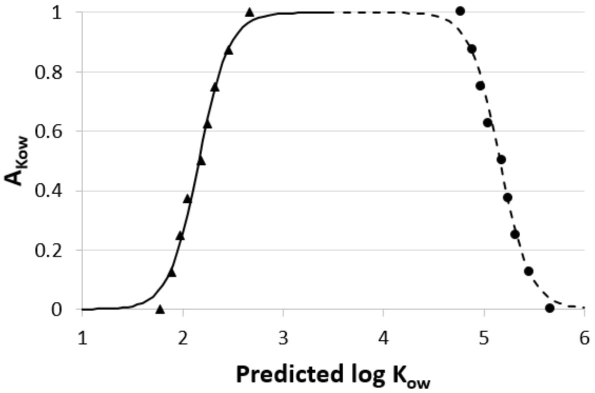

Fig. S7 A_{Kow} as a function of predicted log K_{ow}. The points represent every 10th percentile likelihood of meeting or exceeding the target value between the 10th and 90th percentile. The lines represent sigmoidal functions described by equation S76 (triangles, solid line) and equation S7 (circles, broken line).

$$A_{pKaH} = \frac{1}{1+\left(\frac{9.06}{pK_{aH1}-0.0339}\right)^{107.28}} \quad \text{Eqn. S4}$$

The compounds with A_{pKaH} > 0.5 were evaluated for melting point. The melting points were predicted using the U.S. Environmental Protection Agency's Toxicity Estimation Software Tool (TEST).[15] From these melting points, the melting point acceptability, A_{MP}, was calculated using equation S5, taken from Vanderveen *et al.*[11] This equation gives a quantitative measure of the probability that the predicted melting point for a compound will be less than or equal to 25 °C.

$$A_{MP} = \frac{1}{1+\left(\frac{59.53}{75-MP}\right)^{5}} \quad \text{Eqn. S5}$$

The total performance acceptability, A_{perf}, was calculated for each remaining compound. A_{perf} is the sum of A_{Kow}, A_{pKaH}, and A_{MP}. Compounds with A_{perf} > 2.5 are very likely to act as SHS.

All of the compounds predicted to act as SHS were then evaluated for their risks to the environment and to human health and safety. Seven properties were calculated for each compound using the Toxicity Estimation Software Tool (TEST) developed by the United States Environmental Protection Agency: LC_{50} (fathead minnow, 96 h, "$FMLC_{50}$"), LC_{50} (daphnia magna, 48 h, "$DMLC_{50}$"), LD_{50} (oral, rat), boiling point, flash point, vapour pressure, and bioaccumulation factor (BAF).[15] The first three properties relate to toxicity, the next three properties relate to volatility and flammability, and BAF quantifies the tendency of the compound to bioaccumulate. The acceptability values of these compounds were calculated using equations S6-S11, taken from Vanderveen *et al.* except bioaccumulation acceptability, which is equal to 1 when BAF is less than 500 and equal to 0 when BAF is equal to or greater than 500.[11] The three toxicity acceptability values and the bioaccumulation acceptability value were created to quantify the probability that compounds that meet certain requirements for compounds to be classified as low risk as defined by the globally harmonized system, shown in Table S2.[16] The flash point acceptability value quantifies the probablility that a compound has a flash point above 80 °C, which is higher than the operating temperatures (25-70 °C) of a hypothetical process using SHS described by Vanderveen *et al.*[11]

Table S1 Target values and GHS classifications for the toxicity endpoints used in the virtual screening process.

Property	Target value	GHS Classification[16]
LC_{50} (fathead minnow, 96 h)	>100 mg/L	Not classified as toxic
LC_{50} (daphnia magna, 48 h)	>100 mg/L	Not classified as toxic
LD_{50} (oral, rat)	>2000 mg/L	Category 5: Low acute toxicity but may pose hazard to vulnerable populations
BAF	<500	Low potential to bioaccumulate

The boiling point and vapour pressure acceptabilities quantify the probability that compounds will have sufficiently low volatility to avoid significant inhalation risks and other hazards associated with vapours. The target acceptable boiling point is 180 °C and the target vapour pressure is 0.03 torr as outlined by Vanderveen *et al.*[11] The overall environmental, health, and safety acceptability, A_{EHS}, is the sum of these risk acceptability values and was calculated for each compound. Finally, the compounds were sorted to identify the ones with the highest A_{EHS} scores, compounds that are expected to be SHS with little associated risks. From the list of structures, diamine **4** was chosen as a target molecule for synthesis because of its expected synthetic accessibility.

$$A_{FMLC50} = \frac{1}{1+\left(\frac{73.92}{FMLC_{50}+3.24}\right)^{2.6}} \quad \text{Eqn. S6}$$

$$A_{DMLC50} = 0.0053 \times DMLC_{50} - 0.06 \quad \text{Eqn. S7}$$

$$A_{LD50} = \frac{1}{1+\left(\frac{1255.33}{LD50_{rat}-121.86}\right)^{2.89}} \quad \text{Eqn. S8}$$

$$A_{BP} = \frac{1}{1+\left(\frac{138.8}{BP-38.22}\right)^{35.62}} \quad \text{Eqn. S9}$$

$$A_{FP} = \frac{1}{1+\left(\frac{74.01}{FP-2.87}\right)^{16.73}} \quad \text{Eqn. S10}$$

$$A_{VP} = -1 - 0.6667 \times logVP \quad \text{Eqn. S11}$$

6. References:

1 B. Zou and H.-F. Jiang, *Chin. J. Chem.*, 2008, **26**, 1309–1314.
2 B. Peng, D. Geerdink, C. Farès and N. Maulide, *Angew. Chem. Int. Ed.*, 2014, **53**, 5462–5466.
3 E. G. Knapick, P. Ander and J. A. Hirsch, *Synthesis*, 1985, 58–60.
4 D. D. Reynolds and T. T. M. Laakso, US patent no. 2716134 A, 1955.
5 J. C. Hansen and G. G. I. Moore, US patent no. 5104034 A, 1992.
6 R. S. Davidson and J. W. Goodin, *Eur. Polym. J.*, 1982, **18**, 597–606.
7 K. R. Lassila and J. P. Casey, US patent no. 5371288 A, 1994.
8 J. R. Vanderveen, R. I. Canales, Y. Quan, C. B. Chalifoux, M. A. Stadtherr, J. F. Brennecke and P. G. Jessop, *Fluid Phase Equilibria*, 2016, **409**, 150–156.
9 J. C. Speakman, *J. Chem. Soc.*, 1940, 855–859.
10 A. Albert and E. P. Serjeant, *The determination of ionization constants: a laboratory manual*, Chapman and Hall, London ; New York, 3rd ed., 1984.
11 J. R. Vanderveen, L. Patiny, C. B. Chalifoux, M. J. Jessop and P. G. Jessop, *Green Chem.*, 2015, **17**, 5182–5188.
12 J. R. Vanderveen, J. Durelle and P. G. Jessop, *Green Chem.*, 2014, **16**, 1187–1197.
13 A. Schüller, G. Schneider and E. Byvatov, *QSAR Comb. Sci.*, 2003, **22**, 719–721.
14 A. Schüller, V. Hähnke and G. Schneider, *QSAR Comb. Sci.*, 2007, **26**, 407–410.
14 US EPA, *Toxicity Estimation Software Tool (TEST) v4.1*.
16 *Globally Harmonized System of Classification and Labelling of Chemicals (GHS)*, United Nations, New York and Geneva, 4th edn., 2011.

7. ^{1}H NMR spectra of synthesized compounds

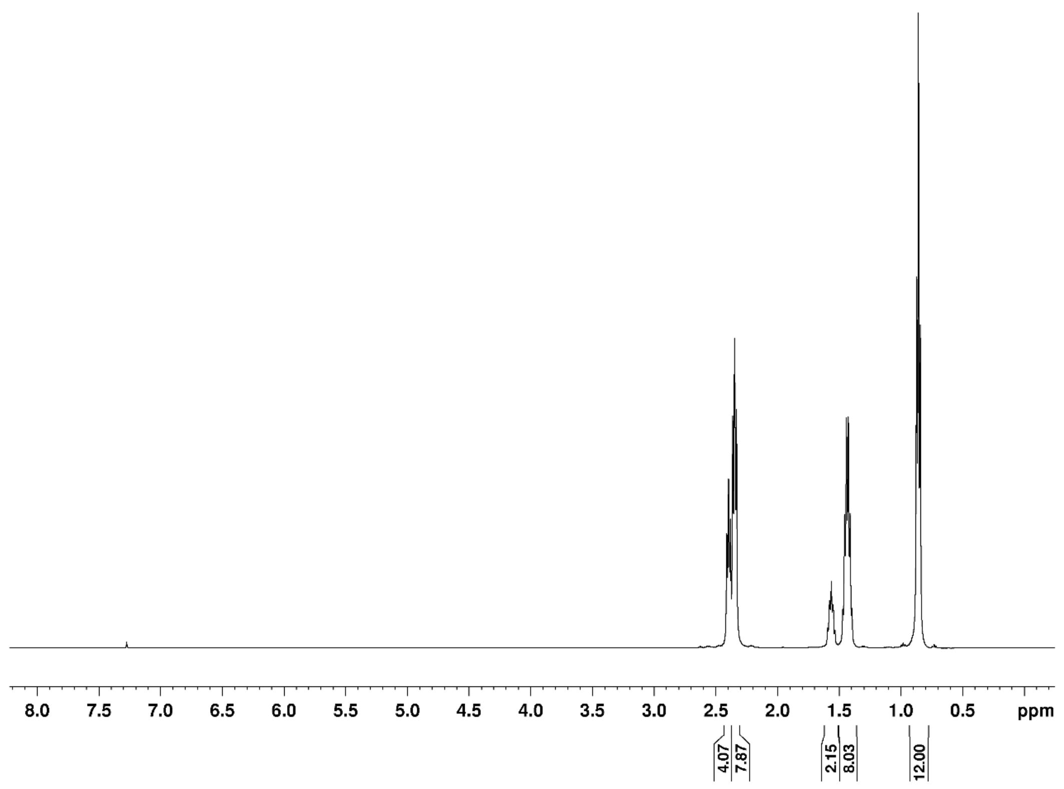

Fig. S8 ^{1}H NMR spectrum of *N*,*N*,*N'*,*N'*-tetrapropyl-1,3-propanediamine (**1**) in chloroform-*d*.

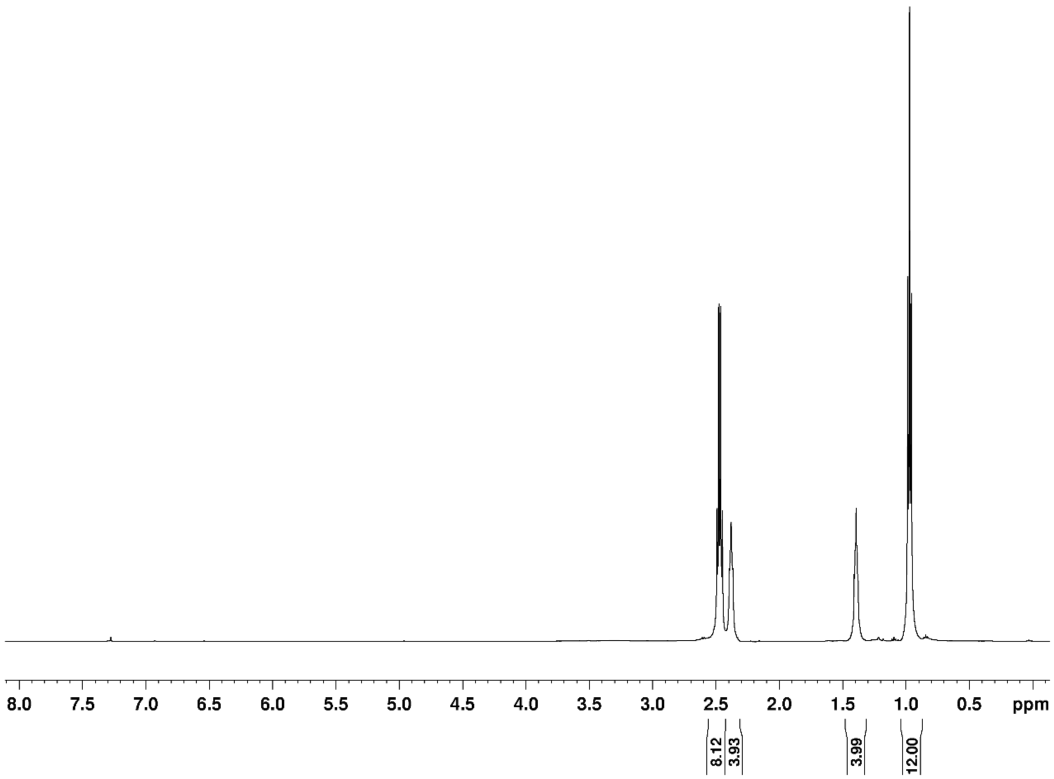

Fig. S9 ^{1}H NMR spectrum of N,N,N',N'-tetraethyl-1,4-butanediamine (**2b**) in chloroform-*d*.

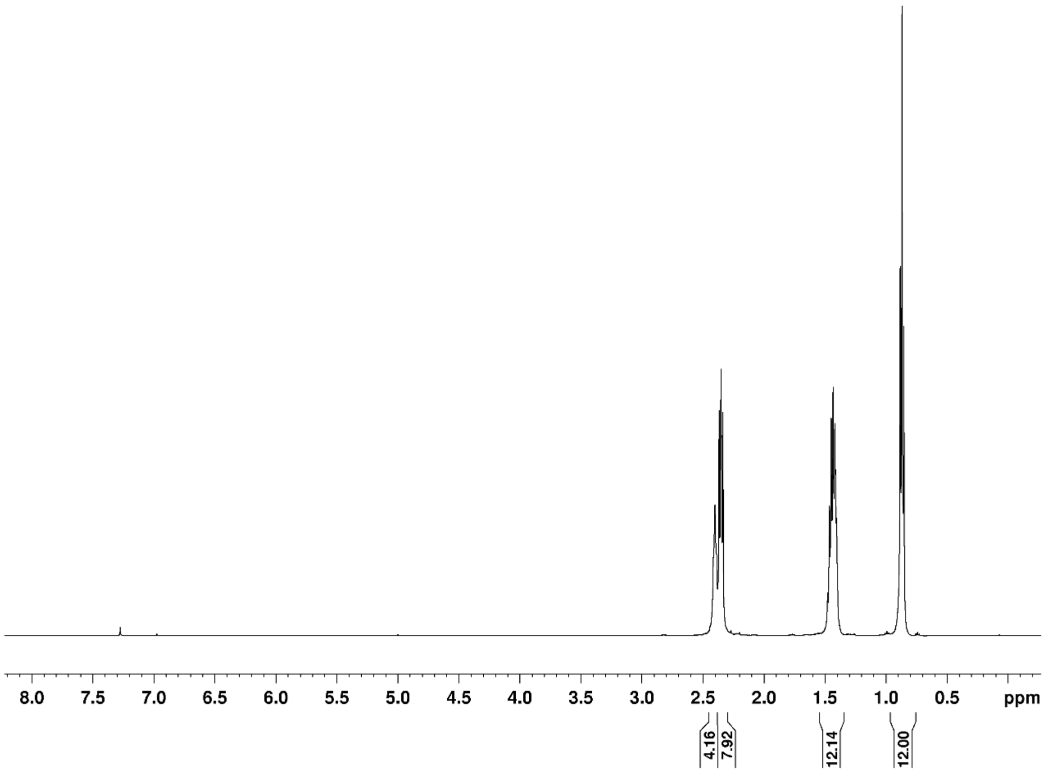

Fig. S10 ^{1}H NMR spectrum of *N,N,N′,N′*-tetrapropyl-1,4-butanediamine (**2c**) in chloroform-*d*.

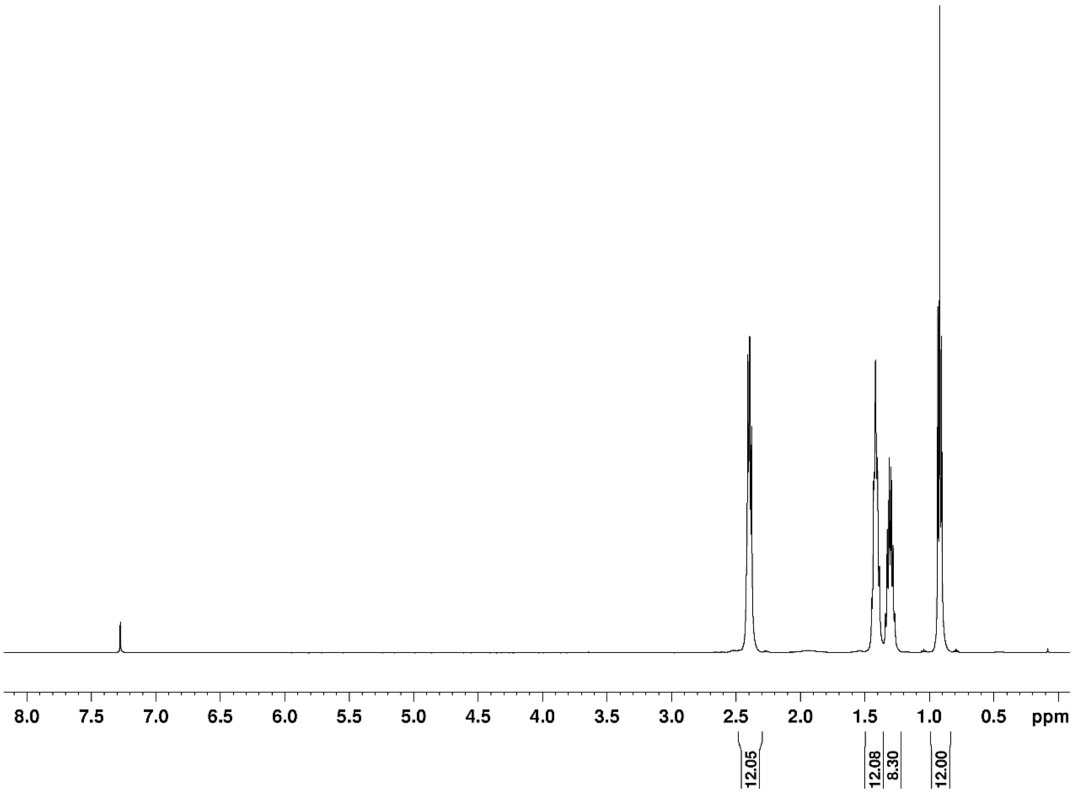

Fig. S11 ^{1}H NMR spectrum of *N*,*N*,*N*′,*N*′-tetrabutyl-1,4-butanediamine (**2d**) in chloroform-*d*.

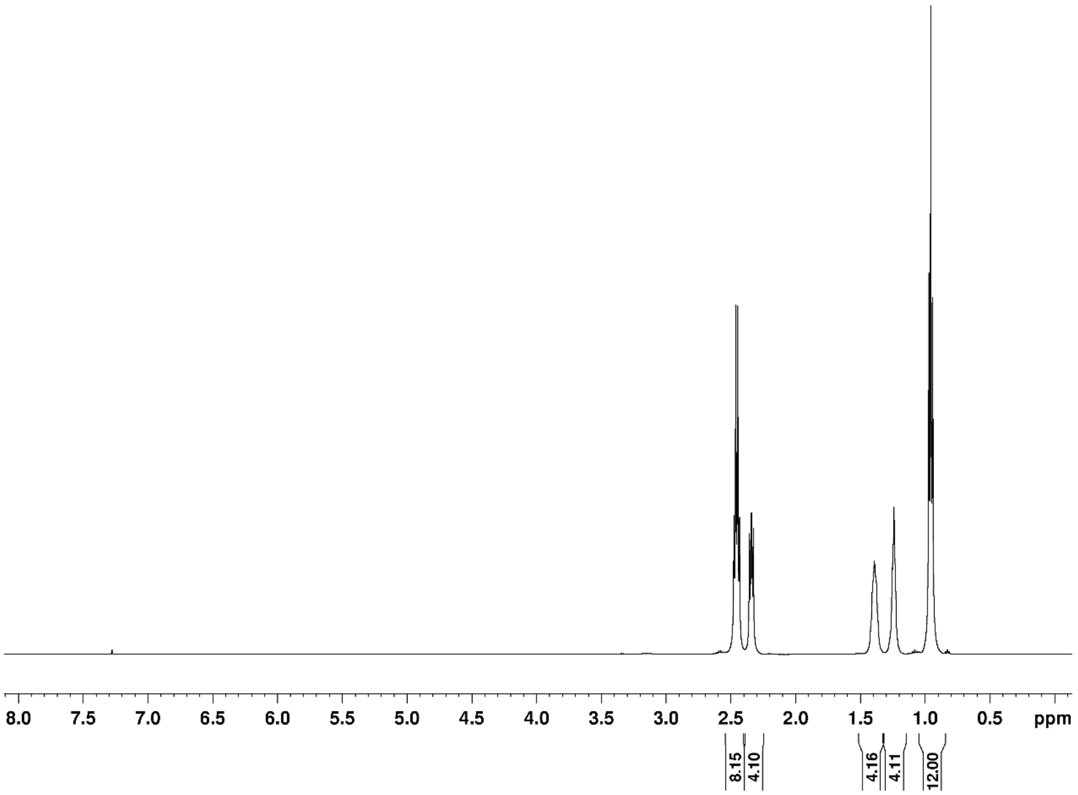

Fig. S12 ¹H NMR spectrum of *N*,*N*,*N'*,*N'*-tetraethyl-1,6-hexanediamine (**3b**) in chloroform-*d*.

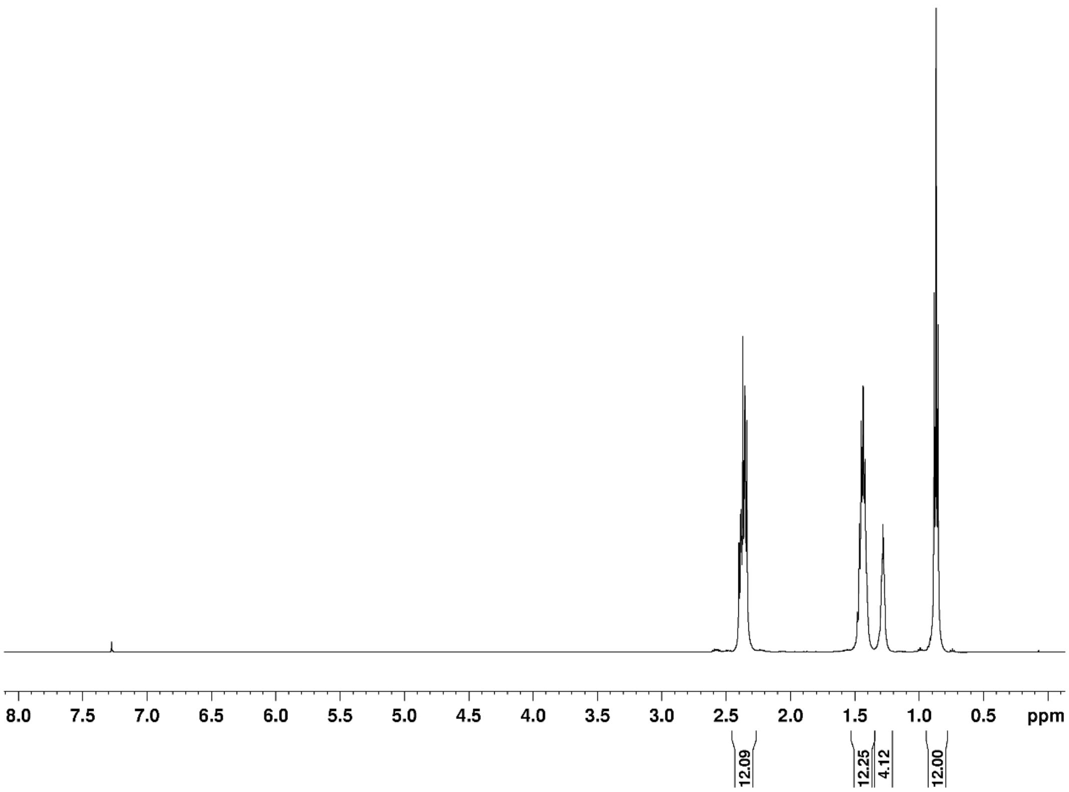

Fig. S13 ^{1}H NMR spectrum of *N*,*N*,*N'*,*N'*-tetrapropyl-1,6-hexanediamine (**3c**) in chloroform-*d*.

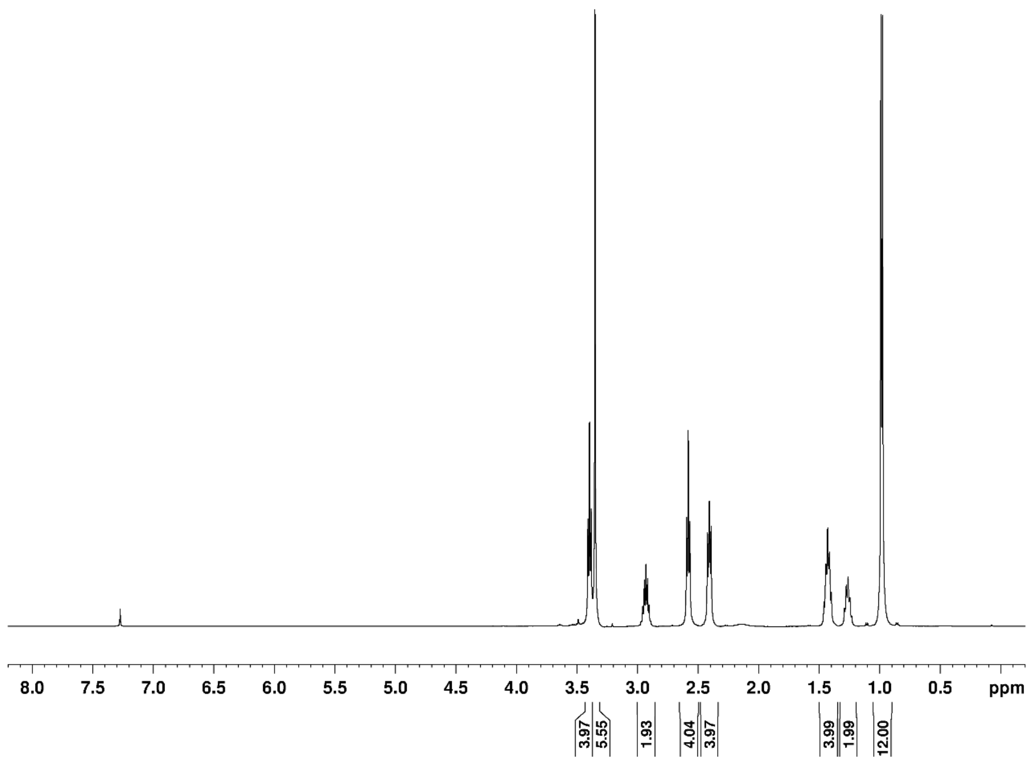

Fig. S14 ^{1}H NMR spectrum of N,N'-dimethoxyethyl-N,N'-diisopropyl-1,5-pentanediamine (**4**) in chloroform-*d*.

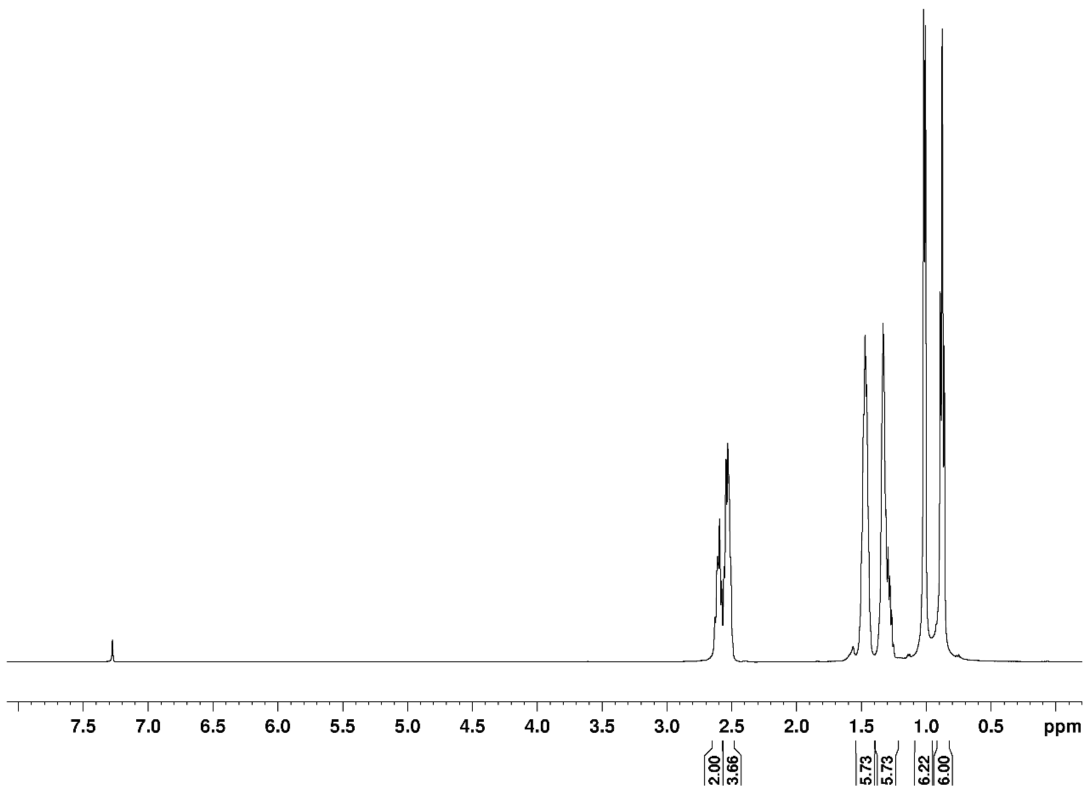

Fig. S15 ^{1}H NMR spectrum of *N,N'*-di-*sec*-butyl-1,6-hexanediamine (**5**) in chloroform-*d*.

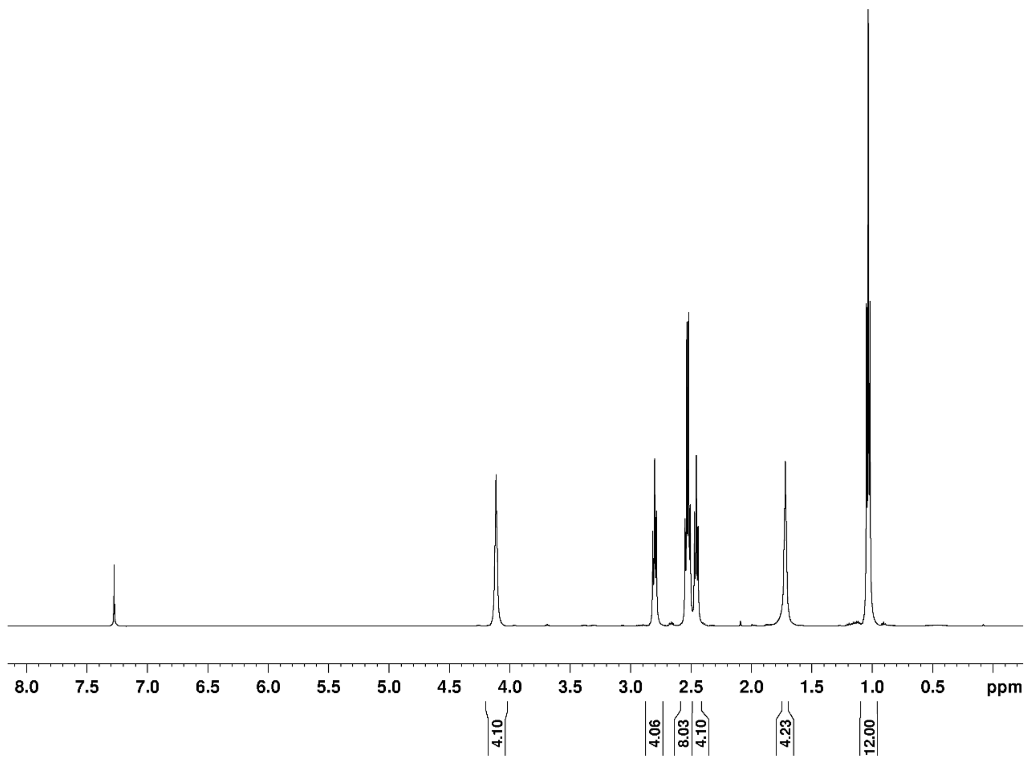

Fig. S16 ^{1}H NMR spectrum of 1,4-butanediol di-(3-(diethylamino)propanoate) (**6a**) in chloroform-*d*.

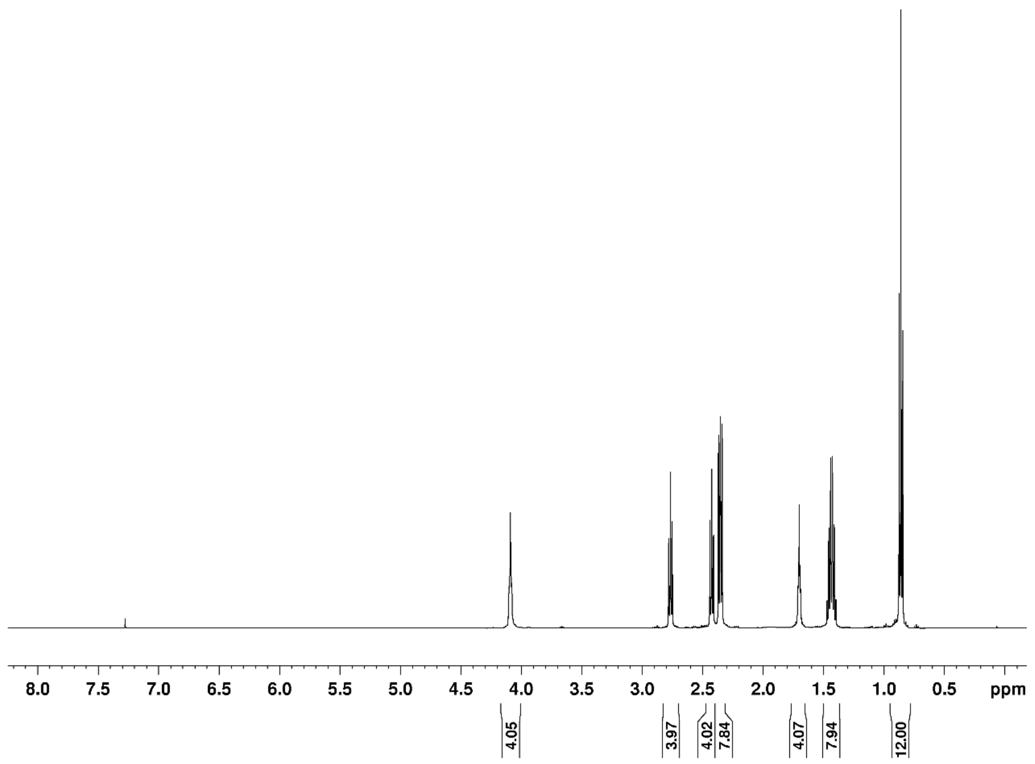

Fig. S17 ^{1}H NMR spectrum of 1,4-butanediol di-(3-(dipropylamino)propanoate) (**6b**) in chloroform-*d*.

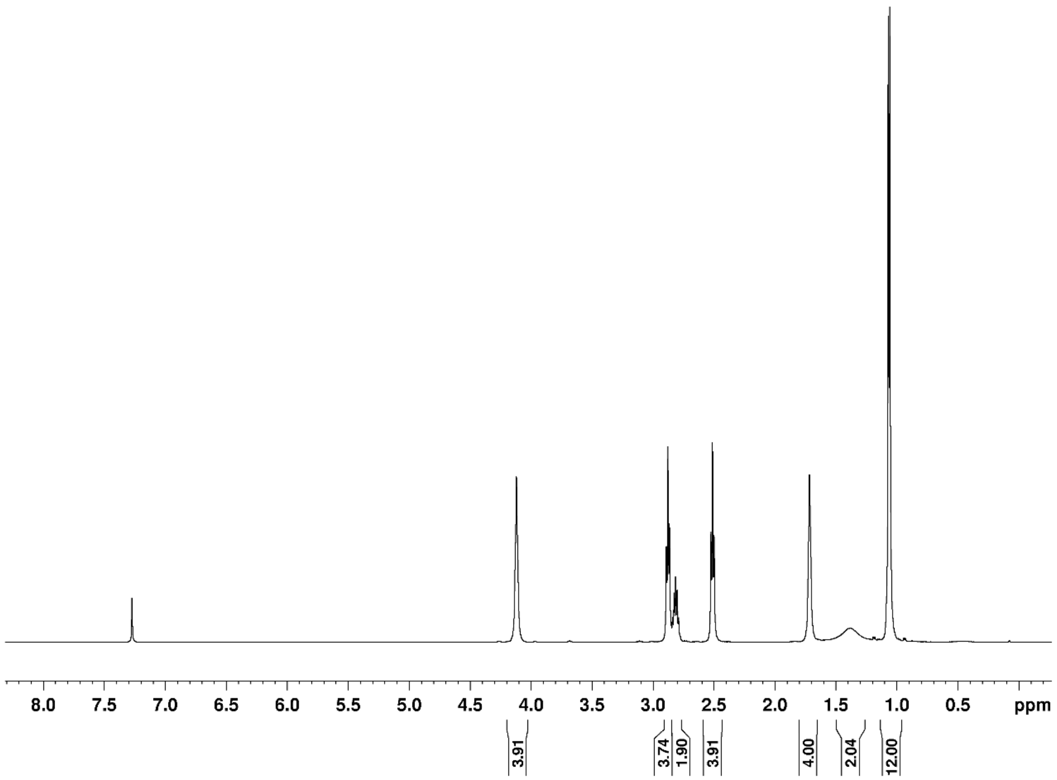

Fig. S18 ^{1}H NMR spectrum of 1,4-butanediol di-(3-(isopropylamino)propanoate) (**7a**) in chloroform-*d*.

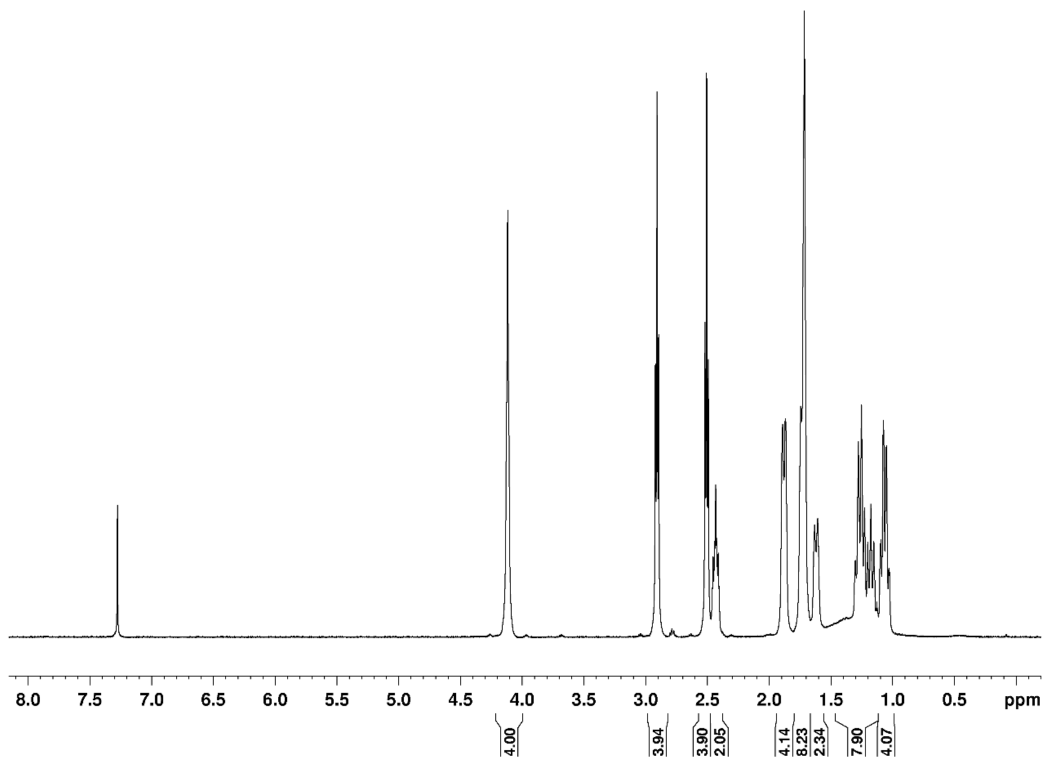

Fig. S19 ^{1}H NMR spectrum of 1,4-butanediol di-(3-(cyclohexylamino)propanoate) (**7b**) in chloroform-*d*.
